# Role of the ACTH-SF1/NR5A1-NRF2 axis in glucocorticoid-induced adrenocortical cell death through mitochondrial ROS accumulation

**DOI:** 10.1038/s41419-026-09306-8

**Published:** 2026-10-29

**Authors:** Naohiro Uchida, Namiko Kawamura, Hironobu Umakoshi, Maki Yokomoto-Umakoshi, Tazuru Fukumoto, Norifusa Iwahashi, Masamichi Fujita, Aya Fujimoto, Kaede Saito, Yuta Hiraike, Fumiya Takahashi, Takashi Baba, Masahide Seki, Yutaka Suzuki, Yoshihiro Ogawa

**Affiliations:** 1 Department of Medicine and Bioregulatory Science, Graduate School of Medical Sciences, Kyushu University, Fukuoka, Japan; 2 Division for Health Service Promotion, The University of Tokyo, Tokyo, Japan; 3 Laboratory for Advanced Research on Pathophysiology of Metabolic Diseases, Graduate School of Medicine, The University of Tokyo, Tokyo, Japan; 4 Department of Anatomy, Kurume University School of Medicine, Kurume, Japan; 5 Department of Molecular Biology, Graduate School of Medical Sciences, Kyushu University, Fukuoka, Japan; 6 Department of Systems Life Sciences, Graduate School of Systems Life Sciences, Kyushu University, Fukuoka, Japan; 7 Department of Computational Biology and Medical Sciences, Graduate School of Frontier Sciences, The University of Tokyo, Chiba, Japan

**Keywords:** Endocrinology, Adrenal gland diseases

## Abstract

Exogenous glucocorticoids are widely used to treat inflammatory and autoimmune diseases, yet the prolonged administration often leads to adrenocortical atrophy and subsequent adrenal insufficiency. However, the molecular mechanisms underlying glucocorticoid-induced adrenocortical atrophy remain incompletely understood. Here, we show that adrenocortical alterations in dexamethasone (DEX)-treated mice are similar to those in human adrenocortical tissues adjacent to cortisol-producing adenoma. During DEX treatment, marked cell death occurs, being restricted to the adrenocortical zona fasciculata (ZF), as a result of reactive oxygen species (ROS)-mediated mitochondrial dysfunction; the pathological changes are all attenuated by co-administration of adrenocorticotropic hormone (ACTH). In DEX-treated mice, ACTH deficiency impairs nuclear factor E2-related factor 2 (NRF2)-dependent redox homeostasis in ZF cells. Activation of the cAMP-PKA signaling pathway, a downstream signaling of ACTH stimulation, increases gene expression of steroidogenic factor 1 (SF1; NR5A1) in cultured adrenocortical cells in vitro, where it binds directly to the *Nrf2* promoter and enhances its transcription, thereby promoting antioxidant gene expression to maintain redox homeostasis. This study highlights the role of the ACTH-SF1-NRF2 axis in glucocorticoid-induced adrenocortical cell death through mitochondrial ROS accumulation, thereby providing a clue to a new therapeutic strategy to prevent or treat secondary adrenal insufficiency during glucocorticoid excess.

## Introduction

The adrenal gland plays a critical role in the integration of the stress response. The outer part of the adrenal gland, or adrenal cortex, consists of three functionally and structurally distinct zones in humans; the zonae glomerulosa, fasciculata, and reticularis (ZG, ZF, and ZR), and secretes a range of steroid hormones in a layer-specific manner; mineralocorticoids (aldosterone), glucocorticoids (cortisol), and adrenal androgens, respectively [1–3].

Endogenous glucocorticoid secretion from the adrenal cortex is tightly regulated by pituitary-derived adrenocorticotropic hormone (ACTH), the production of which is stimulated by corticotropin-releasing hormone from the hypothalamus. On the other hand, glucocorticoids, which possess potent anti-inflammatory and anti-allergic properties, have been widely used for the treatment of autoimmune diseases, allergic conditions, and even cancer [4, 5]. However, prolonged use of glucocorticoids triggers negative feedback on the hypothalamic-pituitary-adrenal axis, thereby reducing endogenous glucocorticoid secretion from the adrenal cortex, and eventually leading to secondary adrenal insufficiency. Similarly, in patients with cortisol-producing adrenocortical adenoma (CPA), pituitary secretion of ACTH is suppressed via a negative feedback mechanism, which is accompanied by the atrophy of non-tumorous tissues adjacent to the CPA as well as of the contralateral adrenal tissues, often leading to secondary adrenal insufficiency after tumor resection [6, 7].

The functional recovery of the atrophied adrenal cortex in all forms of secondary adrenal insufficiency requires a significant amount of time, posing a substantial clinical challenge, as glucocorticoid replacement is often required [8–10]. However, since human adrenocortical tissue is rarely available, how they become atrophied and functionally deteriorated in response to glucocorticoid excess, and how they can be effectively recovered or even prevented, are insufficiently studied. On the other hand, despite marked species differences in adrenocortical biology [11], rodents such as mice have provided a unique experimental model system with which to investigate the mechanisms underlying the functional and structural alterations of the adrenal cortex during glucocorticoid excess [12–17].

Mitochondria have been implicated in a variety of biological processes [18–20]. It is noteworthy that mitochondrial reactive oxygen species (ROS) accumulation damages mitochondrial DNA and reduces mitochondrial membrane potential, thereby leading to cytochrome c release and initiation of the apoptotic pathway [21]. Although glucocorticoid excess exacerbates adrenocortical cell apoptosis [15, 22, 23], whether mitochondrial ROS accumulation is involved in glucocorticoid-induced adrenocortical cell death has not been addressed, and if so, how it might induce adrenocortical cell death is ill-defined.

Here, we show marked adrenocortical atrophy, specifically in the ZF, in dexamethasone (DEX)-treated mice. Our data suggest that ZF cells develop mitochondrial dysfunction and undergo ROS-dependent cell death after DEX treatment. The pathophysiologic alterations are mostly reversed by co-administration of ACTH, which restores the nuclear factor E2-related factor 2 (NRF2)-dependent antioxidant synthesis to maintain cellular redox homeostasis in adrenocortical cells. Interestingly, steroidogenic factor 1 (SF1; NR5A1), a key steroidogenic transcription factor, which is enhanced by ACTH stimulation, directly increases *Nrf2* expression. This study highlights the role of the ACTH-SF1-NRF2 axis in glucocorticoid-induced ZF cell death through mitochondrial ROS accumulation, thereby offering a new therapeutic strategy to prevent or treat secondary adrenal insufficiency during glucocorticoid excess.

## Results

### DEX-induced adrenocortical alterations in mice

**Fig. 1:**
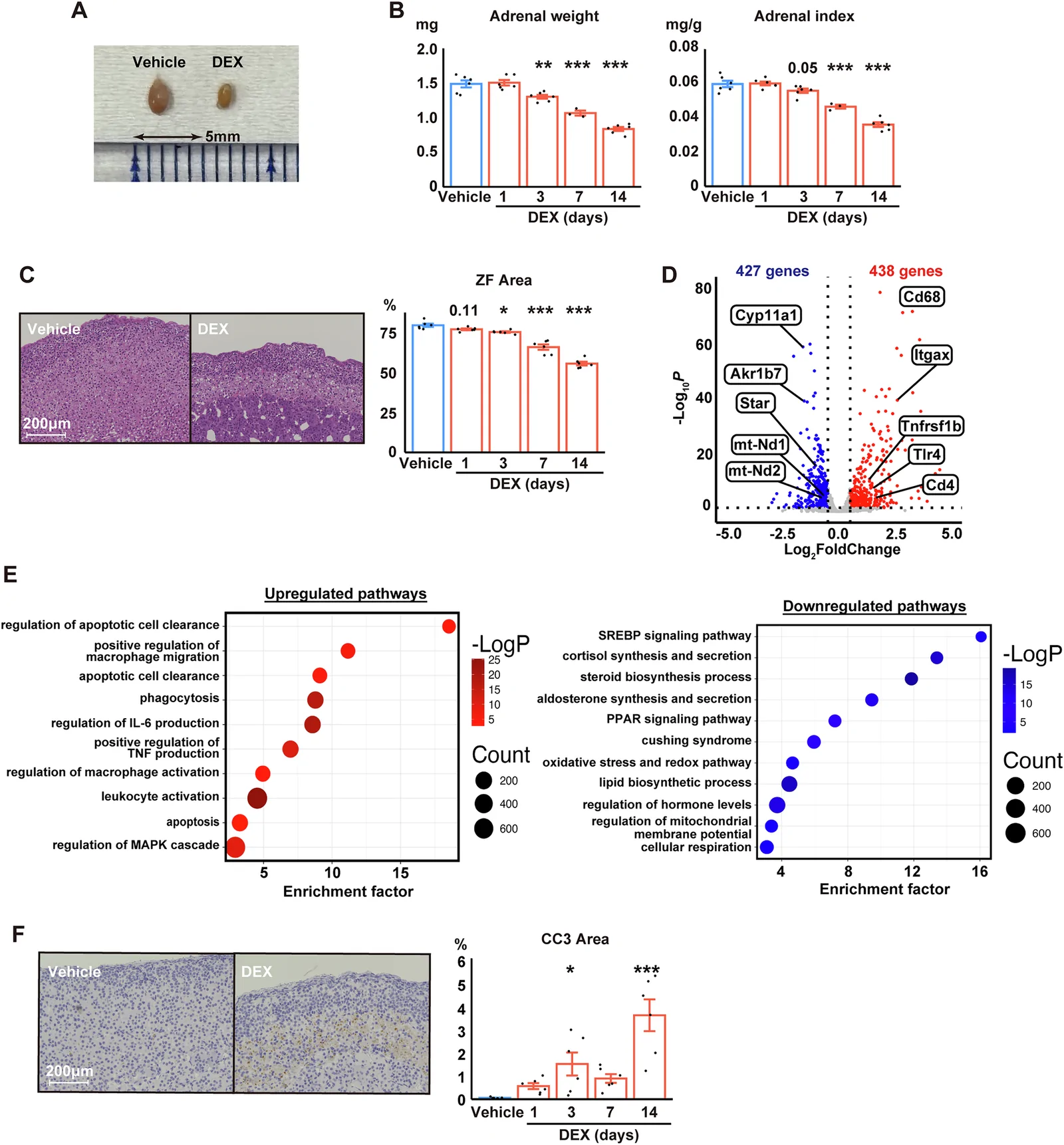
Dexamethasone (DEX) treatment induces adrenal atrophy accompanied by apoptosis and inflammation in the adrenocortical zona fasciculata (ZF). **A** Gross morphology of adrenal glands harvested from DEX- and vehicle-treated mice. **B** Adrenal weight and adrenal index (adrenal weight/body weight) in DEX- and vehicle-treated mice (*n* = 6 mice per group; both adrenal glands per mouse were combined for analysis). Each data point represents the mean value of both adrenal glands from one mouse. **C** Hematoxylin and eosin (HE) staining of adrenal glands from DEX- and vehicle-treated mice. Representative images are shown on the left. Quantification of the ZF area (as a percentage of the total cortical area) is shown on the right (*n* = 6 mice per group). Each data point represents an individual mouse. **D** Volcano plot of differentially expressed genes (DEGs) identified by RNA sequencing (RNA-seq) analysis. Genes upregulated in DEX-treated group are shown in red, and downregulated genes in blue. **E** Gene Ontology enrichment analysis (GO analysis) of DEGs from RNA-seq analysis. Enriched pathways among upregulated genes (left) and downregulated genes (right) following DEX treatment. The x-axis represents the enrichment factor of each pathway. The color intensity indicates the adjusted *P* value, with darker red denoting higher statistical significance. The size of the circles corresponds to the number of DEGs associated with each pathway. **F** Immunohistochemical (IHC) analysis for cleaved caspase-3 (CC3) in the adrenal cortex. Representative images are shown on the left. Brown DAB staining indicates CC3-positive areas. Quantification of CC3-positive area (as a percentage of the cortical area) is shown on the right (*n* = 6 mice per group). Each data point represents an individual mouse. All error bars represent SEM. *P < 0.05, ***P < 0.001 vs. vehicle-treated mice.

To explore how the adrenal cortex becomes atrophied in response to exogenous glucocorticoid excess, we examined the effect of DEX treatment on the adrenal cortex of 8-10-week-old male mice. In this study, plasma corticosterone and ACTH concentrations were markedly suppressed at 1 day of DEX treatment (Fig. S1A). Treatment with DEX resulted in a significant reduction in adrenal weight 3 days after treatment (Fig. 1A, B). In immunohistochemical (IHC) analysis, β-catenin-positive area was defined as the ZG, and the remaining cortical area was defined as the ZF [14]. We found that adrenocortical atrophy was restricted to the ZF during DEX treatment (Fig. 1C and S1B). The expression of steroidogenic genes, such as *Star*, *Cyp11a1*, and *Cyp11b1*, was markedly suppressed in the adrenal glands of DEX-treated mice relative to vehicle-treated mice (Fig. S1C).

To further characterize glucocorticoid-induced adrenocortical atrophy, we conducted RNA sequencing (RNA-seq) analysis of the adrenal glands from mice treated with DEX for 14 days. Principal component analysis (PCA) and the Pearson correlation coefficient matrix of transcriptomic profiles distinguished between DEX- and vehicle-treated mice (Fig. S1D and S1E). In DEX- relative to vehicle-treated mice, we identified 427 downregulated differentially expressed genes (DEGs), including steroidogenic genes such as *Cyp11a1* and *Star*. On the other hand, 438 genes were upregulated, including those associated with inflammation and immune cell markers (*Cd68* and *Itgax*) (Fig. 1D and Supplementary Table 1). Gene Ontology enrichment analysis (GO analysis) revealed that pathways related to steroidogenesis and mitochondrial function are downregulated, while those related to macrophage migration, inflammatory response, and apoptotic cell clearance are upregulated (Fig. 1E).

In IHC analysis, the number of CD68-positive cells, a common marker of macrophages, increased in the ZF 7 days after DEX treatment (Fig. S1F). We also observed that the number of cleaved caspase-3 (CC3) positive cells, a marker of apoptosis, increased specifically in the ZF 3 days after DEX treatment (Fig. 1F).

Sex-specific differences in adrenocortical structure and function have been well recognized [2, 16, 24]. We additionally examined whether DEX induced similar alterations in adrenocortical cells in female mice as those observed in male mice. DEX treatment effectively suppressed plasma corticosterone and ACTH concentration following DEX treatment (Fig. S2A). DEX treatment also induced ZF atrophy and reduced expression of steroidogenic genes (Fig. S2B–S2D). IHC analysis showed that the number of CC3-positive and CD68-positive cells increased following DEX treatment (Fig. S2E and S2F). However, these alterations were less pronounced in female mice than in male mice.

### Adrenocortical alterations in patients with chronic glucocorticoid excess

**Fig. 2:**
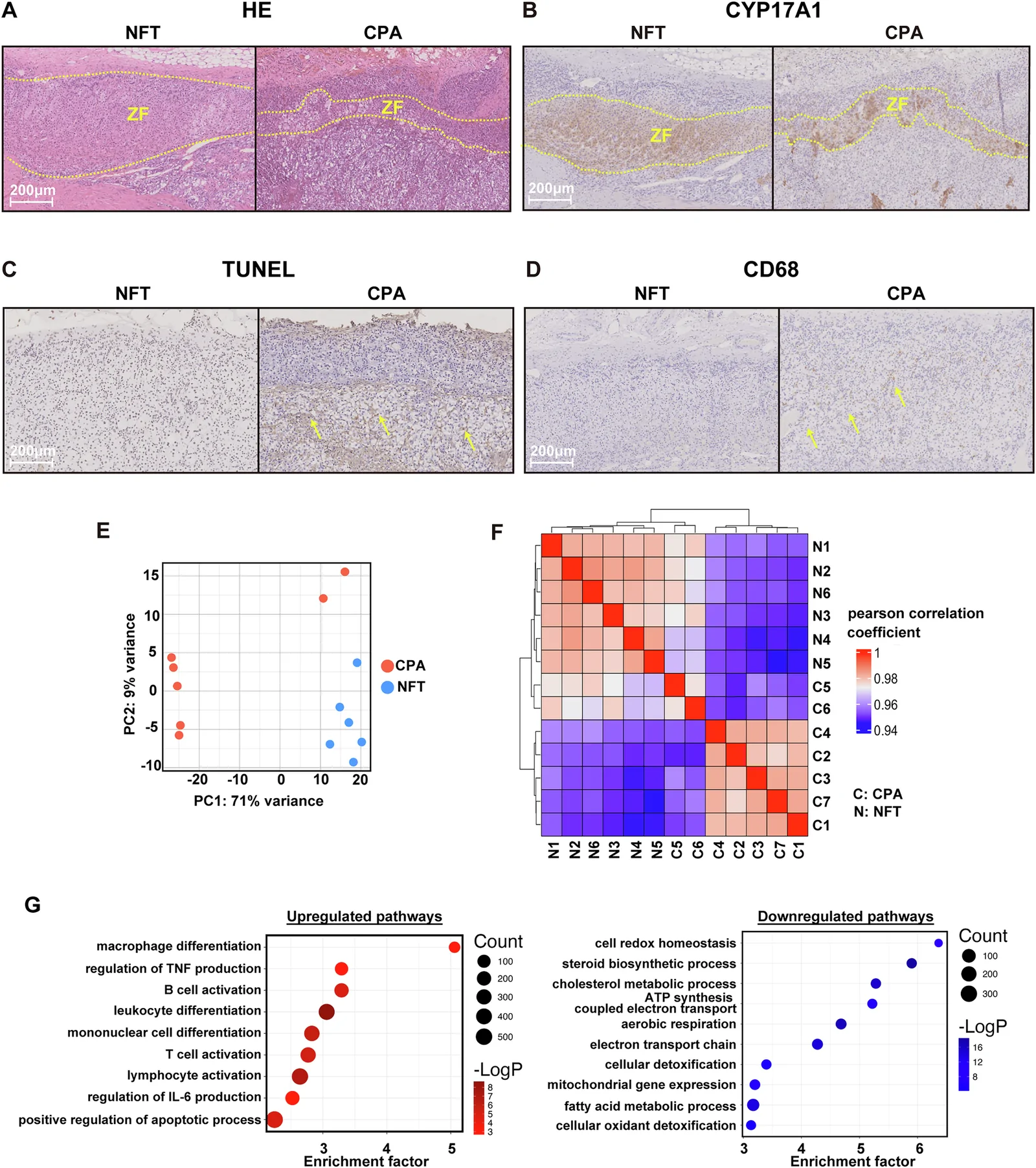
Histological and transcriptomic characterization of human adrenal cortex adjacent to cortisol-producing adrenocortical adenoma (CPA) or non-functioning tumor (NFT). **A**–**D** Histological analysis of adrenal tissues adjacent to NFT (left panels) and CPA (right panels). **A** HE staining showing ZF atrophy in CPA-adjacent adrenal cortex. The ZF area is outlined with yellow dashed lines. **B** IHC for CYP17A1, a marker of ZF, showing reduced ZF thickness in CPA-adjacent tissues. **C** TUNEL staining for apoptotic cells. Yellow arrows indicate DAB-positive apoptotic nuclei in CPA-adjacent tissues. **D** IHC for CD68, a macrophage marker. Yellow arrows indicate DAB-positive macrophage infiltration in CPA-adjacent tissues. **E** Principal component analysis (PCA) of bulk RNA-seq data showing separation between CPA and NFT groups based on transcriptomic profiles. **F** Pearson correlation coefficient matrix of gene expression profiles from individual adrenal samples in the CPA (C1-7) and NFT (N1-6) groups. **G** GO analysis of DEGs between CPA and NFT groups. Upregulated pathways in CPA-adjacent tissues are shown on the left and downregulated pathways are shown on the right.

To explore the effect of chronic glucocorticoid excess on the human adrenal cortex, we compared human adrenocortical tissues adjacent to CPA (CPA group) to those adjacent to non-functioning tumor (NFT) (NFT group). Histologically, the ZG was only maintained, while the ZF— defined as the CYP17A1-positive area, one of the steroidogenic enzymes involved in cortisol synthesis—was markedly atrophied in the CPA group (Fig. 2A, B). In adrenocortical tissues adjacent to CPA, the number of dead cells identified by TUNEL staining increased, accompanied by massive macrophage infiltration, compared with those adjacent to NFT (Fig. 2C, D). We also analyzed our previously published RNA-seq dataset (accession number: E-MTAB-13109) comparing the CPA and NFT groups [25]. PCA and Pearson correlation analyses distinguished the transcriptomic profiles of the CPA group from those of the NFT group (Fig. 2E, F). We also identified the upregulation of the genes and pathways related to macrophage infiltration, apoptosis, and phagocytosis in the CPA relative to NFT group. Conversely, those related to steroidogenesis and mitochondrial function were downregulated (Fig. 2G and Supplementary Table 2).

### Mitochondrial dysfunction in ZF cells from DEX-treated mice

**Fig. 3:**
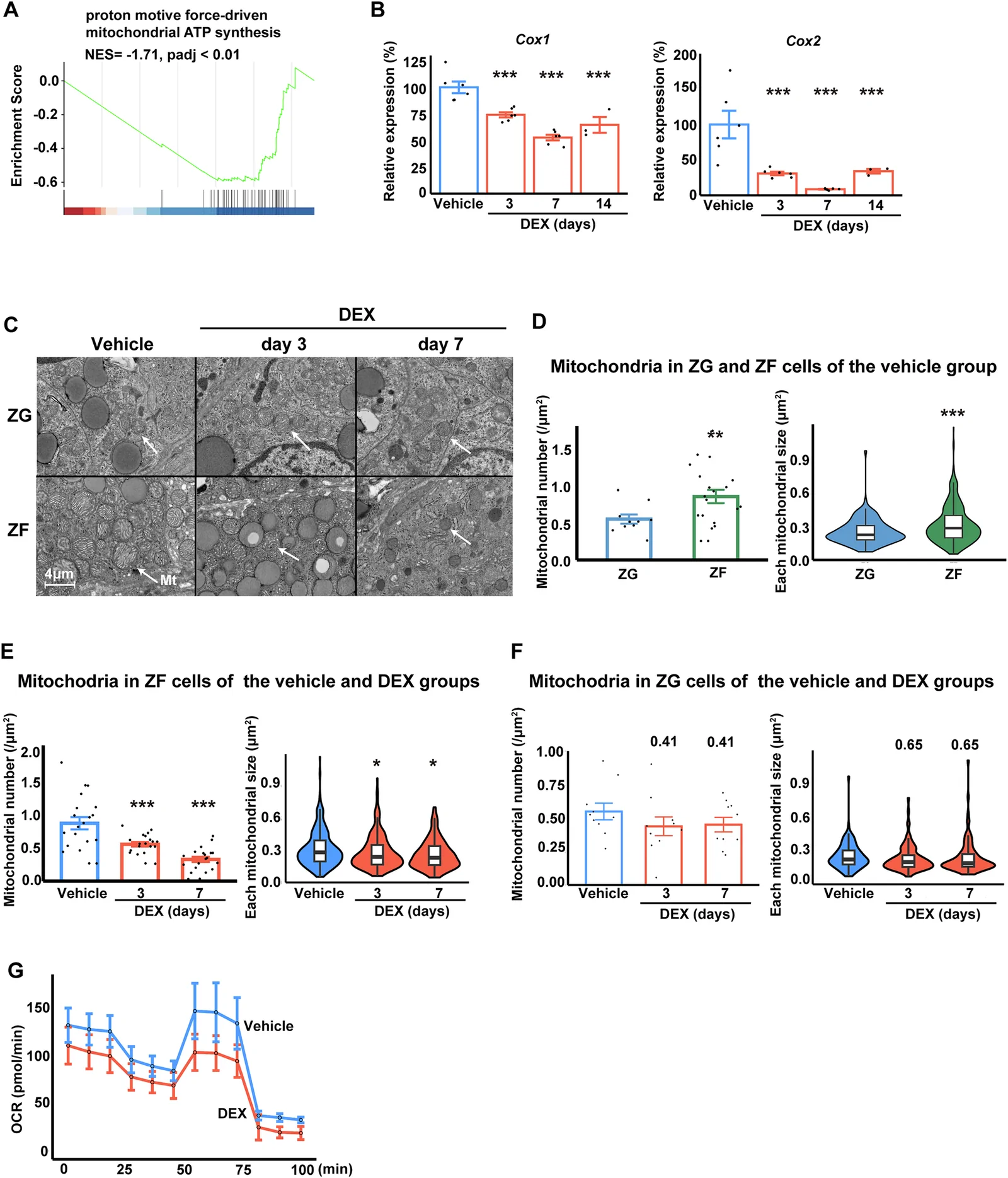
DEX treatment induces morphological and functional mitochondrial dysfunction in the ZF cells of adrenal glands. **A** Gene Set Enrichment Analysis (GSEA) comparing adrenal transcriptomes between DEX- and vehicle-treated mice. The positive normalized enrichment score (NES) indicates the enrichment of genes whose expression is upregulated in the DEX-treated adrenal glands. **B** Gene expression of mitochondrial complex components (*Cox1* and *Cox2*) in adrenal glands from DEX- and vehicle-treated mice measured by reverse transcription quantitative polymerase chain reaction (RT-qPCR) (*n* = 3–6 per group). Expression levels are shown relative to the vehicle-treated group. ***P < 0.001 vs. vehicle-treated mice. **C** Representative transmission electron microscopy (TEM) images of zona glomerulosa (ZG) and ZF cells from DEX- and vehicle-treated mice. White arrows indicate mitochondria. **D** Quantification of mitochondrial number (left) and individual mitochondrial size (right) in ZG and ZF cells from vehicle-treated mice. Mitochondrial number was quantified from 10 images of ZG cells and 20 images of ZF cells. Mitochondrial size was measured for 129 mitochondria in ZG cells and 401 mitochondria in ZF cells from the same images used for mitochondrial number quantification. Each data point represents an individual image (left). **P < 0.01, ***P < 0.001. **E** Quantification of mitochondrial number and size in ZF cells from DEX- and vehicle-treated mice. Mitochondrial number was quantified from 20 images in the vehicle-treated group, 20 images in the 3-day DEX-treated group, and 20 images in the 7-day DEX-treated group. Mitochondrial size was measured for 401 mitochondria in the vehicle-treated group, 256 mitochondria in the 3-day DEX-treated group, and 151 mitochondria in the 7-day DEX-treated group from the same images used for mitochondrial number quantification. Each data point represents an individual image (left). *P < 0.05, ***P < 0.001 vs. vehicle-treated mice. **F** Quantification of mitochondrial number and size in ZG cells from DEX- and vehicle-treated mice. Mitochondrial number was quantified from 10 images in the vehicle-treated group, 10 images in the 3-day DEX-treated group, and 10 images in the 7-day DEX-treated group. Mitochondrial size was measured for 129 mitochondria in the vehicle-treated group, 103 mitochondria in the 3-day DEX-treated group, and 106 mitochondria in the 7-day DEX-treated group from the same images used for mitochondrial number quantification. Each data point represents an individual image (left). **G** Oxygen consumption rate (OCR) of primary adrenocortical cells isolated from DEX- and vehicle-treated mice. All error bars represent SEM.

Evidence suggests the role of mitochondrial dysfunction in the regulation of apoptotic cell death [21]. The adrenal cortex is a steroidogenic organ rich in mitochondria, with the mitochondrial genes and pathways being downregulated after DEX treatment (Fig. 1D, E). We, therefore, focused on adrenal mitochondria as a mediator of ZF cell death in DEX-treated mice. In this study, Gene Set Enrichment Analysis (GSEA) comparing DEX- to vehicle-treated mice revealed that the key mitochondria-related pathways were negatively correlated with DEX treatment (Fig. 3A and Supplementary Table 3). Reverse transcription quantitative polymerase chain reaction (RT-qPCR) analysis confirmed that expression of mitochondrial complex genes (*Cox1* and *Cox2*) is suppressed after DEX treatment (Fig. 3B).

To gain insight into mitochondrial dysfunction in the adrenal cortex from DEX-treated mice, we conducted transmission electron microscopy (TEM) analysis. Without DEX treatment, the size and number of mitochondria were greater in ZF cells than in ZG cells (Fig. 3C, D). Three and 7 days after DEX treatment, we found a substantial reduction in mitochondrial size in ZF cells, with abnormal ultrastructural features, such as cristae malformation and hypodense compartments, all of which are indicative of damaged mitochondria (Fig. 3C, E) [26–31]. In contrast, the size and number of mitochondria in ZG cells remained unchanged after DEX treatment (Fig. 3F).

We next examined whether DEX treatment affects mitochondrial respiratory function through the direct measurement of oxygen consumption rate (OCR) of isolated primary adrenal cells using a Seahorse XF Analyzer. We found that the OCR of the adrenal cells remained impaired after 3 days of DEX treatment (Fig. 3G).

Given the close functional interaction between mitochondria and the endoplasmic reticulum (ER), we next evaluated ER stress in the adrenal glands following DEX treatment [32, 33]. TEM analysis did not reveal morphological features suggestive of ER stress, such as ER dilation or cisternal swelling in ZF cells following DEX treatment [34, 35] (Fig. S3A). In our RNA-seq dataset, ER stress-related pathways were not upregulated in the adrenal glands following DEX treatment (Fig. 1E and Supplementary Table 3). Consistently, the protein levels of phosphorylated eIF2α, a common ER stress marker, were not changed by DEX treatment in the adrenal glands (Fig. S3B). These results indicate that DEX treatment does not induce a detectable ER stress response in the adrenal glands.

### ROS-dependent cell death in adrenal cortex from DEX-treated mice

**Fig. 4:**
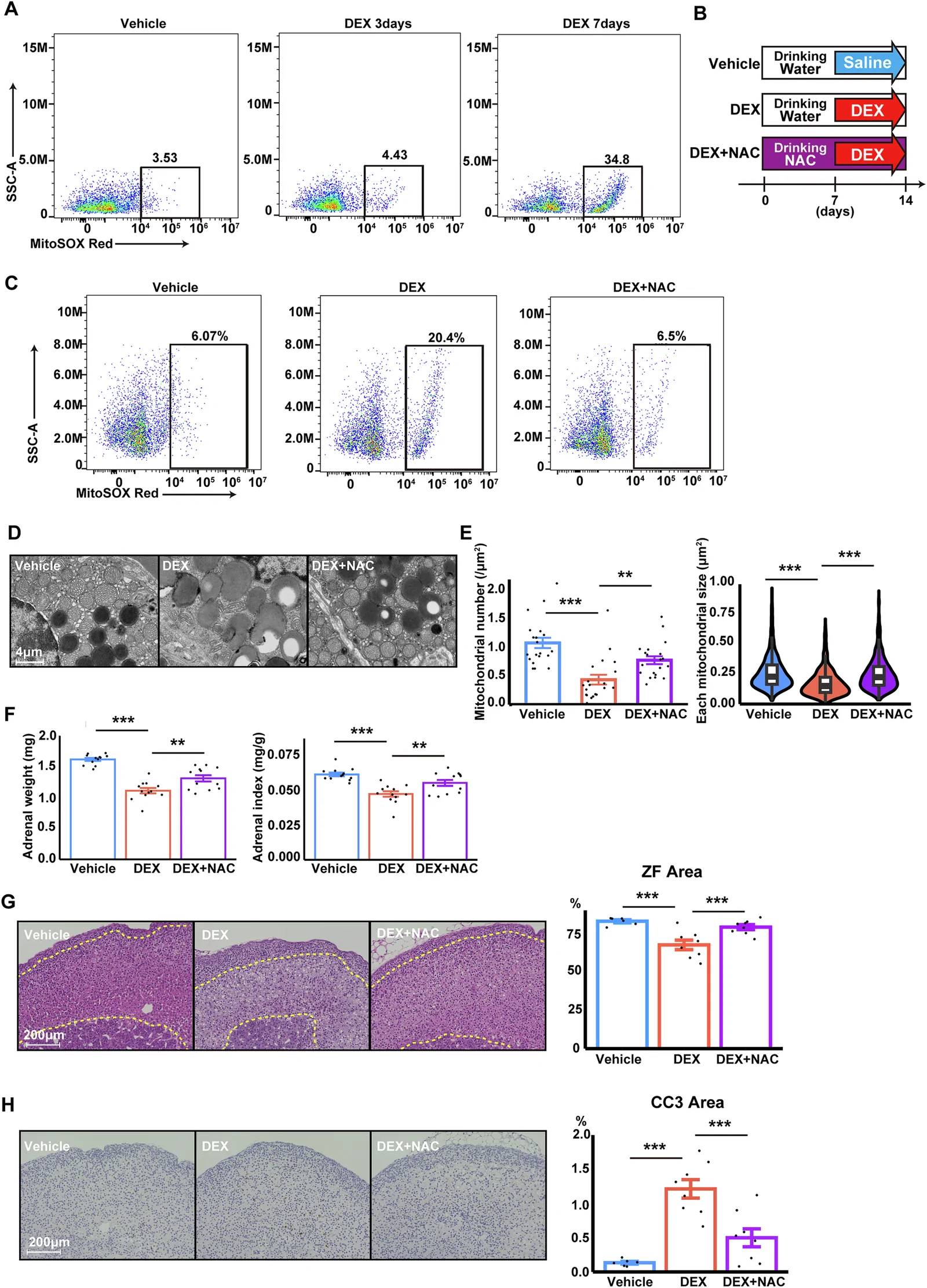
N-acetyl-L-cysteine (NAC) administration alleviates DEX-induced mitochondrial dysfunction, reactive oxygen species (ROS) accumulation, and adrenal atrophy. **A** Representative flow cytometry analysis of mitochondrial ROS levels in isolated adrenocortical cells from DEX- and vehicle-treated mice. Cells were stained with MitoSOX Red and gated on singlets and live cells. Similar results were obtained in 3 independent experiments. **B** Schematic of the antioxidant therapy protocol. **C** Representative flow cytometry analysis of mitochondrial ROS levels in isolated adrenocortical cells from DEX-, DEX + NAC-, and vehicle-treated mice. Cells were stained with MitoSOX Red and gated on singlets and live cells. Similar results were obtained in 3 independent experiments. **D** Representative TEM images of adrenal glands from DEX-, DEX + NAC-, and vehicle-treated mice. **E** Quantification of mitochondrial number and individual mitochondrial size in the ZF of DEX-, DEX + NAC-, and vehicle-treated mice. Left: number of mitochondria per unit area; Right: individual mitochondrial size. Mitochondrial number was quantified from 20 images in the vehicle-treated group, 20 images in the DEX-treated group, and 20 images in the DEX + NAC-treated group. Mitochondrial size was measured for 510 mitochondria in the vehicle-treated group, 206 mitochondria in the DEX-treated group, and 367 mitochondria in the DEX + NAC-treated group from the same images used for mitochondrial number quantification. Each data point represents an individual image (left). **F** Adrenal weight and adrenal index (adrenal weight/body weight) in DEX-, DEX + NAC-, and vehicle-treated mice (*n* = 12 per group; both adrenal glands per mouse were combined for analysis). Each data point represents the mean value of both adrenal glands from one mouse. **G** HE staining of adrenal glands from DEX-, DEX + NAC, and vehicle-treated mice. Representative images are shown on the left. Quantification of the ZF area relative to the total cortical area is shown on the right (*n* = 6–8 per group). Each data point represents an individual mouse. **H** IHC for CC3 in the adrenal cortex of DEX-, DEX + NAC-, and vehicle-treated mice. Representative images are shown on the left. Quantification of CC3-positive area (as a percentage of cortical area) is shown on the right (*n* = 6–8 per group). Each data point represents an individual mouse. All error bars represent SEM. **P < 0.01, ***P < 0.001.

There is considerable evidence that ROS accumulation represents a major cause of cellular mitochondrial dysfunction under various pathophysiological conditions [36–39]. We measured mitochondrial ROS accumulation in isolated primary adrenal cells using MitoSOX Red, a fluorogenic ROS indicator dye which specifically targets cellular mitochondria. In this study, there was a time-dependent accumulation of ROS in primary adrenal cells isolated from DEX-treated mice (Fig. 4A). We found that administration of N-acetyl-L-cysteine (NAC), a ROS scavenger, reduced ROS accumulation and mitigated mitochondrial morphological abnormalities in the adrenal glands of DEX-treated mice (Fig. 4B–E). Importantly, NAC treatment also rescued DEX-induced adrenal weight loss (Fig. 4F). The decreased expression of steroidogenic genes and increased expression of inflammatory genes in DEX-treated mice were attenuated by NAC treatment (Fig. S4A). Histological analysis revealed that NAC attenuated ZF atrophy, cell death, and macrophage infiltration induced by DEX treatment (Figs. 4G, H, and S4B).

### Limited contribution of ferroptosis to DEX-induced adrenocortical cell death

Ferroptosis is a form of non-apoptotic, iron-dependent regulated cell death characterized by intracellular iron accumulation and ROS-mediated lipid peroxidation [40]. The adrenal glands are rich in lipids required for steroid hormone production. Based on our finding that ROS accumulation was a key trigger of DEX-induced cell death in the adrenal glands, we considered the possibility that ferroptosis might contribute to DEX-induced adrenocortical cell death. Quantification of lipid peroxidation showed no apparent change following 7 days of DEX treatment (Fig. S5A). DEX treatment did not increase Fe^2+^ or Fe^3+^ levels in the adrenal glands (Fig. S5B). Administration of ferrostatin-1 did not significantly attenuate DEX-induced adrenocortical cell apoptosis, suppression of steroidogenesis, or macrophage infiltration (Fig. S5C–S5G).

To detect non-apoptotic necrotic cell death, we adapted a previously reported method based on co-staining for propidium iodide (PI) and CC3 [41]. In this study, PI-positive and CC3-negative cells were interpreted as non-apoptotic dying cells. We observed numerous PI-positive cells in the adrenal glands following DEX-treatment; however, these cells were largely positive for CC3. In addition, co-administration of ferrostatin-1 did not reduce the number of PI-positive cells in the adrenal glands of DEX-treated mice (Fig. S5H). Given these findings, we concluded the major form of DEX-induced adrenocortical cell death was apoptosis rather than ferroptosis.

### Downregulation of NRF2-dependent redox homeostasis in ZF cells after DEX treatment

**Fig. 5:**
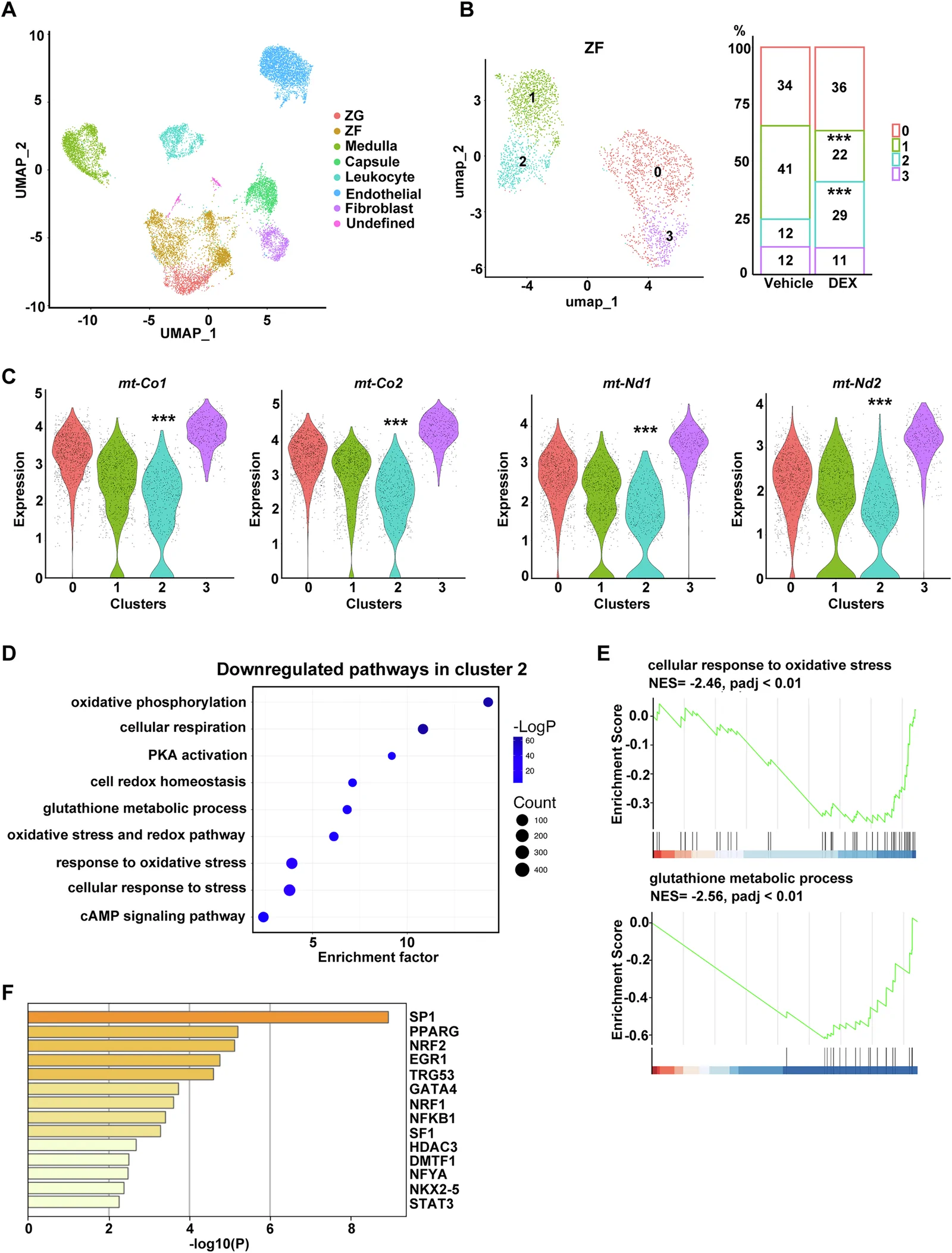
Single-cell transcriptomic analysis reveals that DEX treatment impairs antioxidant gene expression in ZF cells. **A** UMAP plot of single-cell RNA-seq data from adrenal glands of DEX- and vehicle-treated mice. Cell clusters are colored by annotated cell types, including ZG, ZF, medulla, capsule, leukocytes, endothelial cells, fibroblasts, and undefined cells. **B** UMAP plot of re-clustered ZF cells. Cells were grouped into four distinct clusters (0–3). The relative proportion of each cluster is shown for DEX- and vehicle- treated mice on the right. The proportions were analyzed using the chi-squared test, and statistical significance was determined based on adjusted *P* values. ***P < 0.001 vs. vehicle-treated mice. **C** Expression levels of mitochondrial complex genes (*mt-Co1*, *mt-Co2*, *mt-Nd1*, and *mt-Nd2*) across the four ZF clusters. Cluster 2 exhibits the lowest expression of these mitochondrial genes. ***P < 0.001 vs. the other clusters. **D** GO analysis of DEGs in cluster 2. Selected significantly downregulated pathways are shown. **E** GSEA of transcriptomic profiles in cluster 2. The positive NES indicates the enrichment of genes whose expression is upregulated in cluster 2. Antioxidant-related pathways such as cellular response to oxidative stress and glutathione metabolism are significantly suppressed in cluster 2. **F** Transcriptional Regulatory Relationships Unraveled by Sentence-based Text mining (TRRUST) analysis using downregulated DEGs in cluster 2. Transcription factors predicted to be suppressed are ranked by statistical significance.

To explore the molecular mechanism by which ZF cells accumulate ROS, thereby leading to mitochondrial dysfunction after DEX treatment, we performed single-cell RNA seq (scRNA-seq) analysis on the adrenal glands from mice treated with DEX and vehicle for 3 days. The scRNA-seq data, comprising 14,329 cells (5877 and 8452 from DEX- and vehicle-treated mice, respectively), were annotated and clustered using our human adrenal scRNA-seq dataset as a reference (Figs. 5A and S6A) [42]. The proportion of ZF cells was significantly lower in DEX-treated mice than in vehicle-treated mice (Fig. S6B). In this study, steroidogenic genes were downregulated in ZF cells from DEX- relative to vehicle-treated mice, and pathways related to steroidogenesis were also negatively enriched by GSEA analysis (Fig. S6C and Supplementary Table 4). To further elucidate the gene expression profiles of ZF cells, we re-clustered 2,987 ZF cells from the scRNA-seq dataset into four distinct clusters using an unsupervised approach and found that the proportion of cluster 2 was uniquely higher in DEX- relative to vehicle-treated mice (Fig. 5B). Cluster 2 showed the most profound suppression of mitochondrial complex genes relative to the other three clusters, suggesting that it was the ZF subpopulation that was most affected by DEX treatment (Figs. 5C, S6D, and Supplementary Table 5). We next focused on the gene expression profile of cluster 2 and performed GO analysis, demonstrating that the cyclic adenosine monophosphate (cAMP)- protein kinase A (PKA) pathway, a downstream signaling of ACTH, is downregulated in ZF cells in cluster 2 relative to the other three clusters (Fig. 5D). In addition, the downregulated pathways in cluster 2 included those related to mitochondrial function and cellular redox homeostasis, such as glutathione metabolism, which were confirmed by GSEA (Figs. 5D, 5E, S6E, and Supplementary Table 6). We estimated transcription factor activities in cluster 2 using Transcriptional Regulatory Relationships Unraveled by Sentence-based Text mining (TRRUST) analysis (Fig. 5F). We found that the activity of SP1, which is known to regulate a wide range of genes involved in cell growth, differentiation, and stress responses, was suppressed in cluster 2. The activity of NRF2, a key transcription factor involved in glutathione synthesis, was also reduced.

### Attenuation of DEX-induced adrenocortical alterations by co-administration of ACTH

**Fig. 6:**
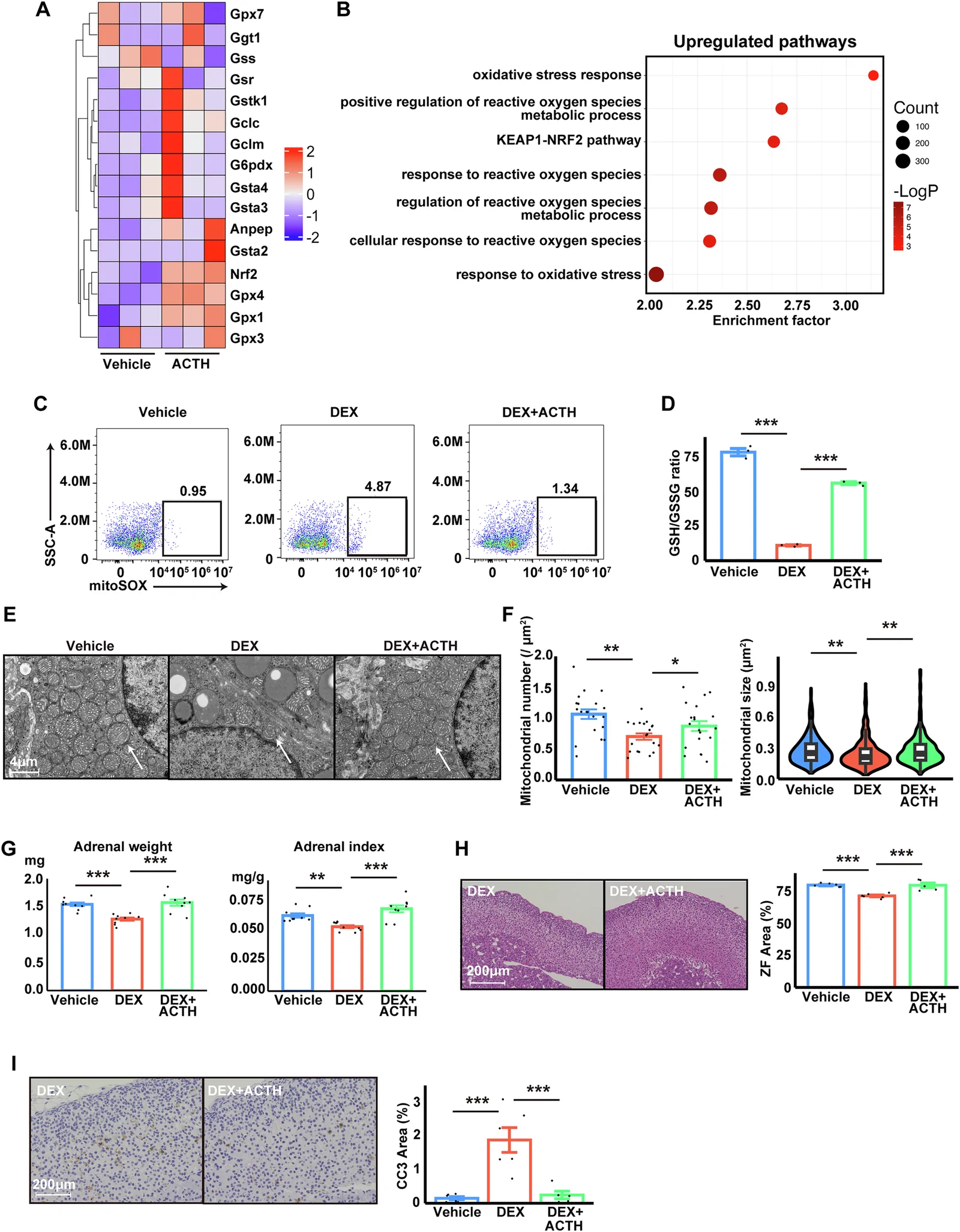
Adrenocorticotropic hormone (ACTH) signaling activates antioxidant pathways and protects against DEX-induced adrenocortical cell death. **A** Heatmap of antioxidant-related gene expression from RNA-seq data of adrenal glands one hour after ACTH or vehicle treatment. **B** GO analysis of DEGs between vehicle- and ACTH- treated mice. Upregulated pathways in ACTH-treated mice are shown. **C** Representative flow cytometry analysis of mitochondrial ROS levels in isolated adrenocortical cells from DEX-, DEX + ACTH-, and vehicle-treated mice. Cells were stained with MitoSOX Red and gated on singlets and live cells. Similar results were obtained in 3 independent experiments. **D** The reduced glutathione (GSH)/oxidized glutathione (GSSG) ratio in adrenal glands (*n* = 3 per group). Each data point represents an individual mouse. **E** Representative TEM images of adrenal glands from DEX-, DEX + ACTH-, and vehicle-treated mice. White arrows indicate mitochondria. **F** Quantification of mitochondrial number and individual mitochondrial size in the ZF of DEX-, DEX + ACTH-, and vehicle-treated mice. Left: number of mitochondria per unit area; Right: individual mitochondrial size. Mitochondrial number was quantified from 20 images in the vehicle-treated group, 20 images in the DEX-treated group, and 20 images in the DEX + ACTH-treated group. Mitochondrial size was measured for 501 mitochondria in the vehicle-treated group, 330 mitochondria in the DEX-treated group, and 410 mitochondria in the DEX + ACTH-treated group from the same images used for mitochondrial number quantification. Each data point represents an individual image (left). **G** Adrenal weight and adrenal index (adrenal weight/body weight) in DEX-, DEX + ACTH-, and vehicle-treated mice (*n* = 9 mice per group; both adrenal glands per mouse were combined for analysis). Each data point represents the mean value of both adrenal glands from one mouse. **H** HE staining of adrenal glands from DEX-, DEX + ACTH-, and vehicle-treated mice. Representative images are shown on the left. Quantification of ZF area relative to the total cortical area is shown on the right (*n* = 6 mice per group). Each data point represents an individual mouse. **I** IHC staining for CC3 in adrenal cortex. Brown DAB staining indicates CC3-positive areas. Quantification of CC3-positive area (as a percentage of the cortical area) is shown on the right (*n* = 6 mice per group). Each data point represents an individual mouse. All error bars represent SEM. *P < 0.05, **P < 0.01, ***P < 0.001.

Because the cAMP-PKA pathway was downregulated along with the downregulation of redox homeostasis pathway (Fig. 5D), we speculated that ACTH stimulation upregulates the redox homeostasis pathway. In this study, we administered ACTH (cosyntropin) alone to mice, and one hour later, plasma corticosterone concentration was elevated (Fig. S7A), when we performed RNA-seq analysis on the adrenal glands collected. PCA distinguished ACTH-treated mice from vehicle-treated mice (Fig. S7B). The expression of several genes involved in glutathione biosynthesis, such as *Gpx4* and *Nrf2*, was elevated in ACTH- relative to vehicle-treated mice, with the redox homeostasis pathway being upregulated by ACTH stimulation (Figs. 6A, 6B, S7C and Supplementary Table 7).

Next, we examined the effect of ACTH administration on DEX-induced adrenocortical alterations. Co-administration of ACTH decreased DEX-induced ROS accumulation in the adrenal glands (Fig. 6C). In addition, the reduced glutathione (GSH)/oxidized glutathione (GSSG) ratio in the adrenal glands decreased after DEX treatment, which was restored by co-administration of ACTH, as assessed by enzymatic colorimetric assay (Fig. 6D). TEM analysis revealed that mitochondrial morphological abnormalities of ZF cells after DEX treatment were ameliorated by co-administration of ACTH, suggesting a restoration of mitochondrial function (Fig. 6E, F). The DEX-induced reduction in adrenal weight was also restored by co-administration of ACTH (Fig. 6G). Suppressed steroidogenic and elevated inflammatory genes expression in the adrenal glands after DEX treatment were also restored by ACTH co-administration (Fig. S7D). Histological analysis revealed that co-administration of ACTH improves ZF atrophy, reduces adrenocortical cell death, and decreases macrophage infiltration (Figs. 6H, 6I, and S7E).

### Increased expression of antioxidant enzymes through the ACTH-SF1-NRF2 axis in adrenocortical cells in vitro

**Fig. 7:**
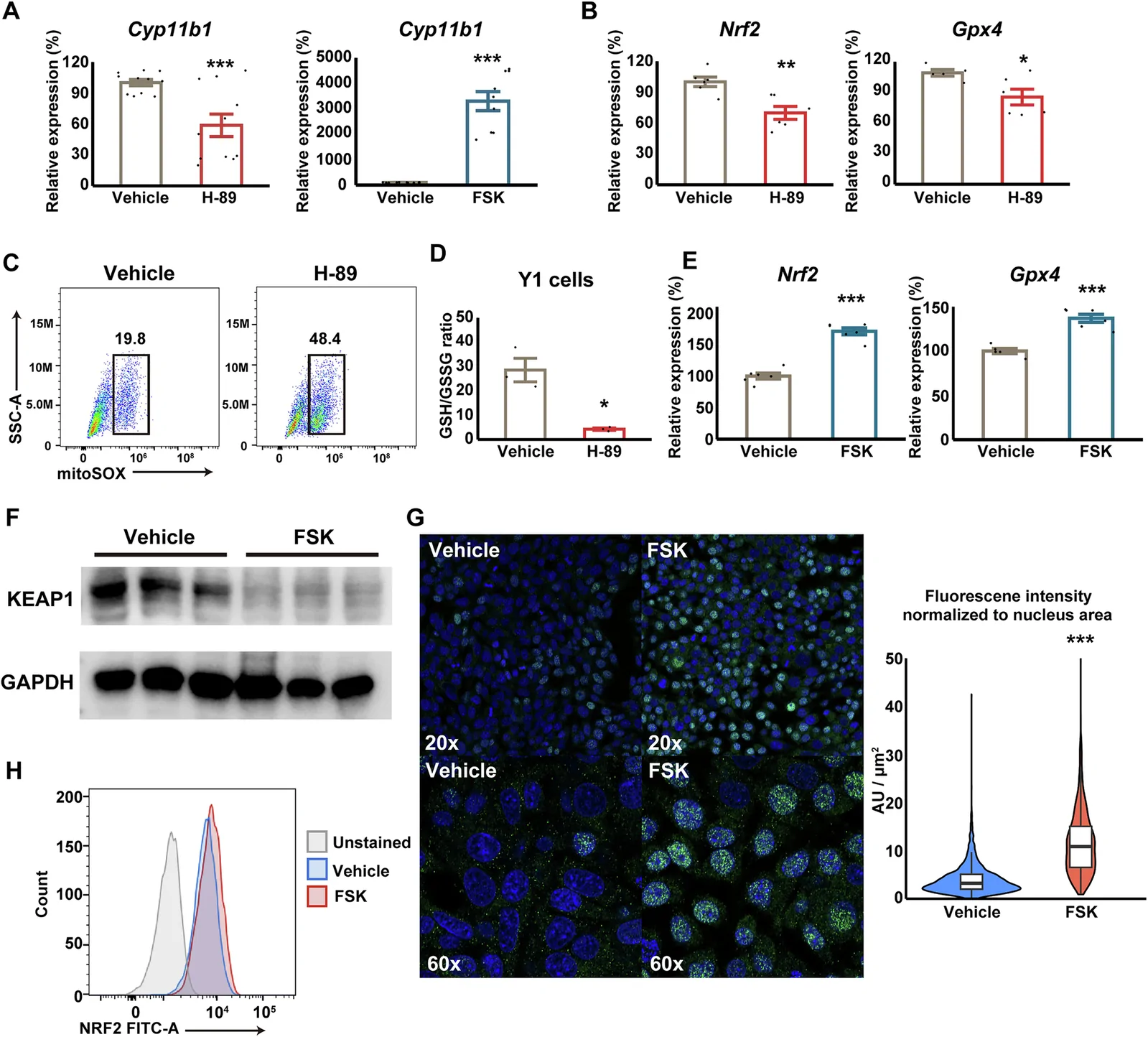
Cyclic adenosine monophosphate (cAMP)- protein kinase A signaling induces nuclear factor E2-related factor 2 (NRF2) activation and antioxidant gene expression. **A** RT-qPCR analysis of *Cyp11b1* expression in Y1 cells following H-89 and forskolin (FSK) treatment. Expression levels are shown relative to the vehicle-treated Y1 cells (*n* = 11 per group). **B** RT-qPCR analysis of expression of antioxidant enzymes (*Nrf2* and *Gpx4*) in Y1 cells following H-89 treatment. Expression levels are shown relative to vehicle-treated Y1 cells (*n* = 5–6 per group). **C** Representative flow cytometric analysis of mitochondrial ROS levels in Y1 cells stained with MitoSOX Red after H-89 treatment. Similar results were obtained in 3 independent experiments. **D** The GSH/GSSG ratio in Y1 cells following H-89 treatment (*n* = 3 per group). **E** RT-qPCR analysis of expression of antioxidant genes (*Nrf2* and *Gpx4*) in Y1 cells following FSK treatment. Expression levels are shown relative to the vehicle-treated Y1 cells (*n* = 5–6 per group). **F** Western blot analysis of Kelch-like ECH-associated protein 1 (KEAP1) protein expression in Y1 cells following FSK treatment, with GAPDH as a loading control. **G** Confocal microscopy images showing the subcellular localization of NRF2 protein in vehicle- and FSK-treated Y1 cells. Representative images at ×20 and ×60 magnification are shown. Quantification of nuclear NRF2 fluorescence intensity is shown on the right. Quantification was performed on cells from 6 images per group (1025 cells in vehicle-treated Y1 cells and 1188 cells in FSK-treated Y1 cells). **H** Y1 cells were stained with an anti-NRF2 antibody after fixation and permeabilization. Histogram shows NRF2 fluorescence intensity in unstained, vehicle-, and FSK-treated Y1 cells. All error bars represent SEM. *P < 0.05, **P < 0.01, ***P < 0.001. Each data point represents an independent biological replicate.

To elucidate how ACTH promotes the redox homeostasis pathway in adrenocortical cells, we performed an in vitro study using the mouse adrenocortical cell line Y1. Since Y1 cells do not express melanocortin-2 receptor (MC2R), we used forskolin (FSK) and H-89, a selective adenylyl cyclase activator and a PKA inhibitor, respectively, to directly activate and inhibit the cAMP-PKA pathway, a downstream signaling of ACTH stimulation. H-89 is known to reduce steroidogenic gene expression [43, 44], also effectively downregulated NRF2-dependent antioxidant enzymes in Y1 cells (Fig. 7A, B). In addition, H-89 treatment induced ROS accumulation and decreased GSH/GSSG ratio in Y1 cells (Fig. 7C, D). On the other hand, FSK treatment upregulated the expression of steroidogenic and NRF2-dependent antioxidant enzyme genes in Y1 cells (Fig. 7A, E). NRF2 is bound to Kelch-like ECH-associated protein 1 (KEAP1) and retained in the cytoplasm, thereby facilitating the ubiquitination and subsequent degradation of NRF2 [45, 46]. In Western blot analysis, KEAP1 levels were downregulated after FSK treatment (Fig. 7F). Using IHC and FACS, we also confirmed that FSK treatment induced nuclear translocation of NRF2, suggesting the activation of NRF2 by FSK (Fig. 7G, H).

**Fig. 8:**
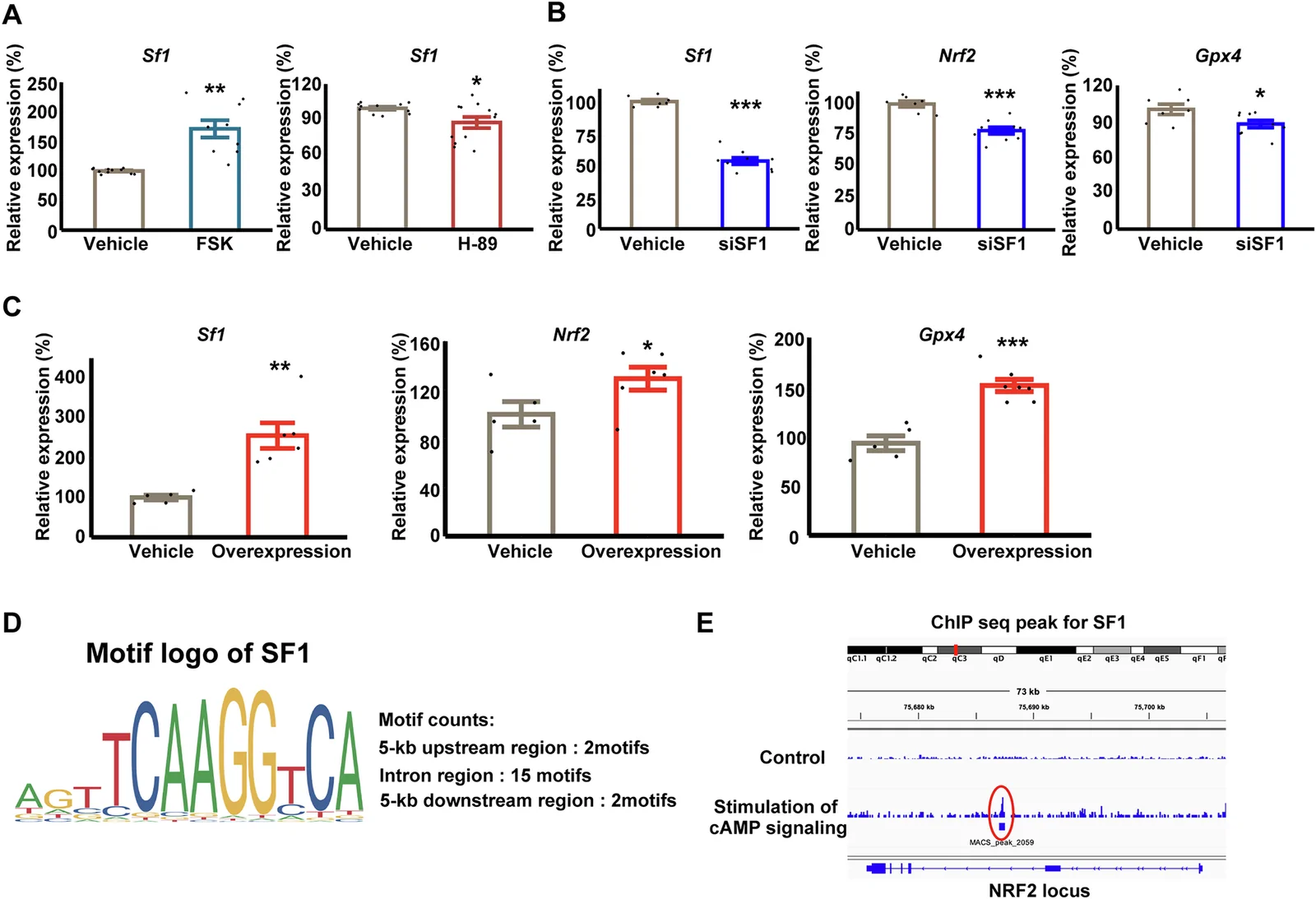
Steroidogenic factor 1 (SF1; NR5A1) binds *Nrf2* locus and positively regulates antioxidant gene expression. **A** RT-qPCR analysis of *Sf1* expression following FSK and H-89 treatment. Expression levels are shown relative to the vehicle-treated Y1 cells (*n* = 10–12 per group). **B** RT-qPCR analysis of expression of *Sf1* and antioxidant genes (*Nrf2* and *Gpx4*) following knockdown of *Sf1* using small interfering RNA (siRNA). Expression levels are shown relative to the vehicle-treated Y1 cells (*n* = 7–9 per group). **C** RT-qPCR analysis of expression of *Sf1* and antioxidant genes (*Nrf2* and *Gpx4*) following *Sf1* overexpression. Expression levels are shown relative to the vehicle-treated Y1 cells (*n* = 5–7 per group). **D** SF1 binding motif sequence. The number and location of predicted SF1 binding motifs within the *Nrf2* locus ( ± 5 kb from the gene) are shown on the right. **E** Public ChIP-seq data for SF1 in Leydig cells after cAMP-mediated activation. A prominent SF1 binding peak is observed in the intragenic region of *Nrf2*. All error bars represent SEM. *P < 0.05, **P < 0.01, ***P < 0.001. Each data point represents an independent biological replicate.

In our scRNA-seq dataset, ZF cluster 2, in which antioxidant gene expression was downregulated after DEX treatment, showed the reduced transcriptional activity of SF1, a critical transcription factor in steroidogenic organs (Fig. 5F). In this study, FSK treatment increased the expression of *Sf1*, whereas H-89 treatment decreased its expression in Y1 cells (Fig. 8A). In addition, knockdown of *Sf1* using small interfering RNA (siRNA) decreased the expression of antioxidant enzymes (Fig. 8B), whereas *Sf1* overexpression in Y1 cells enhanced their expression (Fig. 8C).

Next, we investigated the direct effect of SF1 activity and *Nrf2* expression. There were several SF1-binding motif sequences within the intronic region of *Nrf2* (Fig. 8D). We analyzed the publicly available ChIP-seq data from Leydig cells, which exhibit gene expression profiles similar to those of Y1 cells [47]. Like Y1 cells, Leydig cells are steroidogenic and rely on SF1 to regulate the expression of steroidogenic genes [48, 49]. We identified an SF1-binding peak within the intronic region of *Nrf2* following cAMP stimulation (Fig. 8E).

## Discussion

Glucocorticoid excess, occurring in patients undergoing exogenous glucocorticoid therapy or in those with CPA, results in adrenocortical atrophy. In this study, transcriptomic and histological data revealed that DEX-treatment induced apoptotic cell death in the adrenal glands, which is consistent with a couple of previous reports [50]. A long-term administration of DEX often causes muscle atrophy and osteoporosis, the mechanism of which has been attributed in part to cell death through the direct activation of the glucocorticoid receptor (GR) signaling pathway [51, 52]. However, in our scRNA-seq dataset, gene expression of *Nr3c1*, a protein-coding gene for GR, is lower in adrenocortical cells than in other cell types. In this context, Zheng et al. conducted transcriptomic analysis of adrenals from DEX-treated mice and DEX-treated Y1 cells and found that only a few DEGs are shared between the two groups [12]. Our findings suggest that loss of ACTH signaling—rather than DEX-mediated direct GR activation—is responsible for DEX-induced adrenal pathology, as evidenced by the protective effect of ACTH co-administration in vivo.

In the present study, although our main analyses were conducted in male mice, we also examined DEX-induced alterations in adrenocortical cells in female mice. We found the DEX-induced adrenal atrophy was less pronounced in female mice than in male mice. The milder phenotype in female mice could be attributable, at least in part, to adrenal-intrinsic sexual dimorphism [53, 54]. Further studies are needed to clarify the mechanisms underlying sex-dependent differences in DEX-induced adrenocortical alterations.

We found that DEX treatment causes mitochondrial ROS accumulation and dysfunction in ZF cells. Oxidative stress is characterized by excessive ROS accumulation, which can damage the mitochondrial respiratory chain with reduced mitochondrial membrane potential, thus leading to cellular apoptosis [55]. Mitochondrial dysfunction-associated apoptosis has been recognized as a trigger of numerous acute and chronic diseases, such as neurodegeneration, and metabolic and cardiovascular diseases [56–59]. It has been reported that suppression of cellular ROS accumulation improves a variety of pathologic conditions, such as ischemic diseases of the brain and heart, sepsis, heart failure, and neurodegenerative diseases [60]. Mitochondrial DNA mutations have been implicated in adrenal dysfunction in humans [61], and adrenal mitochondrial dysfunction has been reported to cause adrenal insufficiency in a mouse sepsis model [62]. In this study, NAC, a well-known antioxidant that primarily functions as a precursor to glutathione, effectively restores DEX-induced adrenocortical alterations, suggesting that DEX treatment induced cell death at least in part in a ROS-dependent manner.

Ferroptosis is also considered as a possible mechanism contributing to DEX-induced adrenocortical cell death. Ferroptosis is characterized by the aberrant accumulation of intracellular iron and lipid peroxidation. Because glutathione metabolism and GPX4 activity are central to the suppression of lipid hydroperoxide accumulation, we performed several experiments to determine whether ferroptosis was involved in DEX-induced adrenal dysfunction. Our findings do not support a major contribution of ferroptosis in this model, however, it’s involvement cannot be completely ruled out, as some parameters tended to be partially improved by ferrostatin-1.

While ACTH is well recognized for its role in stimulating steroidogenesis, the data of this study suggest that ACTH-MC2R signaling also regulates the expression of antioxidant enzymes. In this study, we found that DEX treatment alone results in the downregulation of the glutathione synthesis pathway in ZF cells, which is reversed by co-administration of ACTH. It is important to understand the molecular mechanism by which the ACTH-MC2R signaling pathway regulates the expression of antioxidant enzymes. In our in vitro analysis, cAMP-PKA signaling elicited an antioxidant response by modulating the expression of NRF2, a key transcription factor involved in glutathione biosynthesis. These observations suggest that ACTH deficiency during DEX treatment attenuates NRF2-dependent redox homeostasis, thereby promoting ROS accumulation and mitochondrial dysfunction in ZF cells, which ultimately results in adrenocortical cell death.

SF1 is known as a transcription factor that regulates the expression of steroidogenic genes [63, 64]. In addition to its role in steroidogenesis, SF1 also regulates other biological processes [47]. Our findings suggest that SF1 functions as a transcriptional regulator linking the cAMP-PKA signaling pathway to *Nrf2* expression. This study provides the first evidence for the role of SF1 as a regulator of oxidative stress response in steroidogenic organs, revealing a previously unrecognized physiological role for SF1 beyond its well-established function in steroidogenesis.

In conclusion, we demonstrate that ACTH deficiency as a result of DEX treatment causes ROS accumulation and mitochondrial dysfunction in adrenocortical cells through the downregulation of antioxidant enzymes, ultimately leading to cell death. This study represents the first demonstration that the transcription factor SF1 acts downstream of the ACTH-cAMP-PKA signaling pathway and promotes *Nrf2* expression, thereby activating antioxidant genes to protect adrenocortical cells from oxidative stress. Our data suggest that an antioxidant approach provides a clue to a new therapeutic strategy to prevent or treat secondary adrenal insufficiency during glucocorticoid excess.

## Methods

### Animals

Seven-week-old male and female C57BL/6 J mice were purchased from Charles River Laboratories Japan Inc. (Yokohama, Japan). The animals were housed in a temperature-, humidity-, and light-controlled room (12-h light and 12-h dark cycle) and allowed free access to water and standard diet (CRF-1LID10; ORIENTAL YEAST CO., LTD. Tokyo, Japan) at Research Center for Human Disease Modeling, Graduate School of Medical Sciences, Kyushu University (Fukuoka, Japan). After 1- to 2-week acclimation, mice were subjected to the experiments. Mice were randomly allocated to experimental groups. All procedures involving animals were performed in accordance with relevant guidelines and regulations.

### DEX administration

Mice were given water with or without 0.03% (w/w) DEX (Merck KGaA, Darmstadt, Germany) *ad libitum* for 2 weeks to perform bulk RNA-seq analysis. They were anesthetized with isoflurane and a micro-osmotic pump (Alzet Model; 1003D, 1007D or 1002, DURECT Corporation, Cupertino, CA, USA) was implanted subcutaneously between the shoulder blades.

The pump contained DEX dissolved in saline at a dose of 8 μg/day, equivalent to 0.03% (w/w), which was effective in C57BL/6 J mice. In all experiments, mice received subcutaneous injection of an equal volume of saline as a vehicle (vehicle-treated mice).

### NAC administration

NAC (FUJIFILM Wako Pure Chemical Corporation, Osaka, Japan) was dissolved in drinking water at a concentration of 15 mg/mL and orally administered to mice. NAC was administered 7 days before and concurrently during subcutaneous administration of DEX for 7 days.

### ACTH administration

In the short-term experiment, mice were subcutaneously injected with 0.45 mg/kg ACTH (CORTROSYN; Alfresa Pharma Corporation, Osaka, Japan), and samples were collected 1 h later. In the long-term experiment, ACTH (0.5 μg/day) was continuously administered via an osmotic pump during DEX treatment, and samples were collected 3 days later.

### Ferroptosis inhibitor administration

Ferrostatin-1 (1 mg/kg; Cayman Chemical Company, Ann Arbor, MI, USA) was dissolved in dimethyl sulfoxide (DMSO) and diluted with saline to a final concentration of 5% DMSO. Ferrostatin-1 was administered intraperitoneally to mice once daily for 7 days concurrently with DEX treatment. Mice in the DEX and vehicle groups received equal volumes of 5% DMSO intraperitoneally.

### Hormone analysis

Blood samples were collected from the retro-orbital vein using Haematokrit-Kapillaren (Brand GmbH + CO KG, Wertheim, Germany) under isoflurane anesthesia and immediately transferred to collection tubes. The samples were centrifuged at 5000 × *g* for 10 min at 4 °C, and the separated plasma was stored at −80 °C until use. Plasma corticosterone and ACTH levels were measured using a Corticosterone Enzyme Immunoassay Kit (Arbor Assays, Ann Arbor, MI, USA) and a Mouse/Rat ACTH ELISA Kit (Abcam, Cambridge, MA, USA), respectively. Hormone levels were measured in technical duplicates, and the mean values from *n* = 3 to 6 biological replicates are presented.

### Iron assay

Fe²⁺ and Fe³⁺ concentrations in adrenal glands from DEX- or vehicle-treated mice were measured using an Iron Assay Kit (ab83366, Abcam). Four adrenal glands were pooled to generate one sample. The measured Fe²⁺ and Fe³⁺ concentrations were normalized to the total protein content of each sample, which was determined by BCA assay (Pierce^TM^ BCA Protein Assay Kits, Thermo Fisher Scientific) and were measured in technical duplicates. All data are presented as relative expression levels, with the vehicle group set to 100%.

### Histological analysis

The mouse adrenal glands were collected and incubated in 4% paraformaldehyde (PFA) at 4 °C for 3 days, after which the fixative was replaced with 70% ethanol. The tissues were then embedded in paraffin. Paraffin-embedded adrenal specimens were sectioned at a thickness of 5 μm and stained with hematoxylin and eosin (HE). To assess DEX-induced adrenal atrophy, the percentage of ZF area relative to total cortical area was measured. The percentages of CD68-positive and CC3-positive areas were calculated as the ratio of positively stained areas to the cortical area. For each mouse, the entire area of one adrenal section was imaged and used for quantitative analysis. Primary antibodies used for IHC analysis of mouse samples included anti-CD68 (1:400, #ab125212; Abcam) and anti-CC3 (1:100, #9661; Cell Signaling Technology, Danvers, MA, USA).

The detailed clinical information for each tumor was described previously [25]. Paraffin-embedded adrenal specimens were used for IHC analysis. Primary antibodies used for it included anti-CYP17A1 (1:250, #ab134910; Abcam) and anti-CD68 (1:150, #M0876; Agilent Technologies, Dako, Glostrup, Denmark). TUNEL staining was performed according to the manufacturer’s instructions (ab66110; Abcam). All images of tissue sections were captured using a BZ-8000 microscope and analyzed with BZ-II Analyzer (version. 1.31, Keyence, Osaka, Japan) and ImageJ software.

### Necrosis detection assay

Necrotic cells were detected in vivo by PI injection [41]. DEX-, DEX+Ferrostatin1-, or vehicle-treated mice were intravenously administered with PI (25 μg/mL, 200 μL) via tail vain 10 min before sacrifice. Trimmed adrenal glands were collected in ice-cold PBS and embedded in frozen Optimal Cutting Temperature Compound as frozen block. Frozen blocks were sectioned and incubated with an anti-CC3 antibody (1:100, #9661; Cell Signaling Technology) overnight, followed by incubation with a fluorescent secondary antibody (1:1000, #A-11008, Thermo Fisher Scientific) for 60 min. Sections were mounted with DAPI-containing mounting medium. PI-positive and CC3-negative cells interpreted as non-apoptotic and necrotic cells.

### Cell culture experiments

The mouse adrenocortical cell line Y1 (RCB0533; provided by RIKEN BRC, Tsukuba, Japan) was maintained in Dulbecco’s Modified Eagle Medium (DMEM) supplemented with 10% fetal bovine serum (FBS) and 1% penicillin-streptomycin at 37 °C in a humidified atmosphere containing 5% CO₂. Y1 cells were treated with 40 μM H-89 (Selleck Biotechnology, Yokohama, Japan) or 3 μM FSK (Nacalai Tesque, Kyoto, Japan) for 24 h to inhibit or activate the cAMP–PKA signaling pathway, respectively. For SF1 knockdown, Y1 cells were transfected with SF1-targeting siRNA (SASI_Mm01_00031633; Merck KGaA) using FuGENE® SI Transfection Reagent (Promega Corporation, Madison, WI, USA), according to the manufacturer’s instruction, and incubated for 24 h. Y1 cells were also transfected with the vector expressing both GFP and SF1 [65], using FuGENE® Transfection Reagent (Promega Corporation). The GFP-positive cells were sorted by BD FACSMelody^TM^ cell sorter (BD Biosciences, San Jose, CA, USA).

### RT-qPCR analysis

Total RNA was extracted from adrenal glands using ISOGEN (NIPPON GENE, Tokyo,Japan), and reverse-transcribed into cDNA using the PrimeScript™ RT Reagent Kit (TaKaRa, Shiga, Japan). RT-qPCR was performed using THUNDERBIRD^®^ Next SYBR^TM^ qPCR Mix (TOYOBO, Osaka, Japan) on a CFX Connect (Bio-Rad Laboratories, Hercules, CA, USA), according to the manufacturer’s protocol. Gene expression levels were normalized to *36b4* expression and were measured in technical duplicates. Primer sequences are listed in Supplementary Table 8. All data are presented as relative expression levels, with the vehicle group set to 100%.

### Western blot analysis

The mouse adrenal glands and Y1 cells were lysed in radioimmunoprecipitation assay buffer containing a protease inhibitor cocktail (Nacalai Tesque) and phosphatase inhibitors (PhosSTOP, Roche, Basel, Switzerland). Samples were then separated by sodium dodecyl sulfate–polyacrylamide gel electrophoresis (SDS-PAGE) using 4-15% gradient gels (Bio-Rad Laboratories) and transferred to polyvinylidene difluoride (PVDF) membranes. Immunoblotting was performed using anti-Phospho-eIF2 alpha (Ser51) (1:500, #9721, Cell Signaling Technology), anti-eIF2 alpha (1:1000, #9722, Cell Signaling Technology), anti-KEAP1 (1:5000, #8047, Cell Signaling Technology), and anti-rabbit IgG (1:20000, #711-036-152, Jackson ImmunoResearch Laboratories, West Grove, PA, USA). GAPDH (1:10000, #015-25473, FUJIFILM Wako Pure Chemical Corporation) was used as loading control.

### Bulk RNA-seq analysis

Whole adrenal glands were collected from 3 mice per group for bulk RNA-seq analysis. Gene expression data were used as input for the R package DESeq2 (version 1.38.3) [66]. Gene counts were normalized by applying the median-of-ratios method using the sizeFactors and counts functions. DEGs were detected using the results function. The Benjamini-Hochberg method was used to correct for multiple comparisons. DEGs were defined as genes with an absolute log_2_ fold-change greater than 0.5 and an adjusted P-value less than 0.05. GSEA was performed using the R package clusterProfiler (version 4.6.2). GO analysis was performed using Metascape with default parameters [67]. PCA was conducted using the plotPCA function on gene expression data processed with the vst function.

### scRNA-seq analysis

#### Library preparation and sequencing

scRNA-seq was performed with fixed RNA Profiling (10x Genomics, Pleasanton, CA, USA). The adrenal glands from 8 mice per each group were stored in liquid nitrogen. Single cells were isolated from frozen adrenal glands according to the manufacture’s instruction (CG00053: Tissue Fixation & Dissociation for Chromium Fixed RNA Profiling). Libraries were constructed from 1 ×10^6^ cells/sample according to the manufacturer’s instruction (CG000477: Chromium Fixed RNA Profiling Reagent Kits). The libraries were sequenced using NovaSeq6000 (Illumina, Inc., San Diego, CA, USA). Mapping and counting were performed using Cell Ranger (version 7.1.0) with the reference genome mm10.

#### Quality control

For quality control of scRNA-seq data, decontamination of ambient RNA was first performed using the python package CellBender (version 0.3.1) [68]. Then, doublets were removed using the R package scDblfinder with default setting [69]. Cells with more than 10,000 expressed genes were also considered doublet and removed. Cells with less than 500 expressed genes and those with the percentage of mitochondrial gene expression higher than 10% were considered low-quality/dead cells and removed.

#### Normalization, batch correction, and dimensional reduction

After quality control, the scRNA-seq data were processed using the R package Seurat (version 4.3.0) [70]. The unique molecular identifier (UMI) count data were normalized using NormalizeData function. PCA using the RunPCA function was performed with the top 2,000 highly variable genes. Batch-corrected principal component embeddings were calculated using the R package harmony (version 0.1.1) [71]. Uniform Manifold Approximation and Projection (UMAP) was performed using the RunUMAP function with the top 30 batch-corrected principal component embeddings [70]. Unsupervised clustering was performed using the shared nearest-neighbor modularity optimisation-based clustering algorithm with the FindNeighbors and FindClusters functions. The top 30 batch-corrected principal component embeddings were used as input for the FindNeighbors function. The resolution parameter of the FindClusters function was set to 0.3.

#### Differential expression analysis

Differential expression analysis was performed using FindMarkers or FindAllMarkers function with the Wilcoxon rank sum test. *P* values were adjusted for multiple testing with Benjamini-Hochberg correction. DEGs were defined as those with a log fold change >0.25 and an adjusted *P* value < 0.05. GO analysis and GSEA were performed using the same method as the bulk RNA-seq analysis.

### TEM analysis

The adrenal glands were collected and fixed with 2% PFA and 2% glutaraldehyde in 0.1 mol/L PBS at 4 °C for 24 h, washed three times with PBS, and then treated with 2% osmium tetroxide in 0.1 mol/L PBS at 4 °C for 2 h before being subjected to TEM analysis (EM-14830RUBY2; JEOL Ltd., Tokyo, Japan). Data analysis was performed using 10-20 images of view from two mice per each group. The size and number of mitochondria were measured by layer using ImageJ software.

### Primary cultures of adrenocortical cells

The adrenal glands in mice were minced into small pieces and incubated at 37 °C for 25 min in a collagenase digestion solution containing bovine serum albumin (FUJIFILM Wako Pure Chemical Corporation), collagenase (Merck KGaA), and DNase I (Merck KGaA), using a shaking water bath. The resulting single cells were maintained in DMEM with 10% FBS and used for mitochondrial function and ROS assays.

### Mitochondrial function assay

Adrenal glands were collected from 6-8 mice per group and pooled in collagenase-containing medium. After dissection, primary adrenocortical cells (1.5 × 10⁵ cells/well) were immediately seeded into XFe24 cell culture microplates (Agilent Technologies, Santa Clara, CA, USA). Mitochondrial respiration was evaluated using the Seahorse XFe24 Extracellular Flux Analyzer (Agilent Technologies) with the Mito Stress Test Kit (Agilent Technologies). Three baseline measurements of OCR were recorded prior to the sequential injection of mitochondrial inhibitors; oligomycin (500 nM), FCCP (1 μM), rotenone (1 μM), and antimycin A (1 μM) from Thermo Fisher Scientific. Each group included 7-8 biological replicates.

### Measurement of mitochondrial ROS and lipid peroxidation

Isolated adrenocortical cells were collected from 5 mice per group. Mitochondrial ROS levels were quantified using MitoSOX Red fluorescence (Thermo Fisher Scientific) and lipid peroxidation levels were quantified using BODIPY C11 (Thermo Fisher Scientific) by flow cytometry [72]. Primary adrenal cells were stained with 5 μM MitoSOX Red or 2 μM BODIPY C11 together with propidium iodide (PI) for 15 min at room temperature. The stained cells were analyzed using a CytoFLEX flow cytometer (Beckman Coulter, Brea, CA, USA). MitoSOX Red and oxidized BODIPY C11 fluorescence were detected in the PE and FITC channels, respectively. Fluorescence intensity was quantified in PI-negative cells using FlowJo software (BD Biosciences).

### Measurement of GSH and GSSG levels

The levels of GSH and GSSG in adrenal glands and Y1 cells were quantified using the GSSG/GSH Quantification Kit (G257; Dojindo, Kumamoto, Japan) according to the manufacturer’s instructions. Each sample was generated from both adrenal glands of a single mouse. Each ratio was measured in technical duplicates.

### Assessment of NRF2 nuclear translocation

Y1 cells were treated with 3 μM FSK for 24 h, then fixed with 4% PFA for 10 min at room temperature. After fixation, the nuclear membrane was permeabilized with 0.1% Triton X-100 for 10 min. The cells were then incubated with an anti-NRF2 antibody (1:500, #12721; Cell Signaling Technology) for 30 min at room temperature, followed by incubation with a fluorescent secondary antibody (1:1000, #A-11008, Thermo Fisher Scientific) for another 30 min. The stained cells were analyzed using a CytoFLEX flow cytometer or imaged with an LSM 700 confocal microscope (Carl Zeiss, Oberkochen, Germany). Fluorescence intensity was quantified using FlowJo software or ImageJ software.

### Motif scanning for SF1

The SF1 binding motif was identified using the JASPAR database (motif ID: MA1540.3) [73]. Motif scanning around the *Nrf2* locus (from 5 kb upstream to 5 kb downstream of the gene) was performed using FIMO software with default parameters (MEME Suite) [74].

### ChIP-seq data analysis

ChIP-seq data were obtained from a publicly available dataset (GSE106955). The downloaded FASTQ files were mapped to the mouse reference genome (mm10) using Bowtie2 (version 2.5.3) [75]. To identify regions of SF1 enrichment, mapped reads were analyzed using MACS2 (version 2.2.9.1) with default parameters [76].

The cells were then incubated with an anti-NRF2 antibody (1:500, #12721; Cell Signaling Technology) for 30 min at room temperature, followed by incubation with a fluorescent secondary antibody (1:1000, #A-11008, Thermo Fisher Scientific) for another 30 min. The stained cells were analyzed using a CytoFLEX flow cytometer or imaged with an LSM 700 confocal microscope (Carl Zeiss, Oberkochen, Germany). Fluorescence intensity was quantified using FlowJo software or ImageJ software.

### Statistics

Data is expressed as mean ± SEM, and *P* value < 0.05 was considered statistically significant unless otherwise mentioned. Statistical analysis was performed using two-tailed Student’s *t* test and repeated measures two-way ANOVA was performed with Benjamini-Hochberg post hoc test using R. Data were assumed to follow a normal distribution with similar variance between groups. Sample sizes were determined based on previous experience and published studies in the field. No statistical methods were used to predetermine sample size. Samples with insufficient material for analysis were excluded. No blinding was performed during experiments or outcome assessment.

## Supplementary information

Supplementary Figures

Supplementary Table 1

Supplementary Table 2

Supplementary Table 3

Supplementary Table 4

Supplementary Table 5

Supplementary Table 6

Supplementary Table 7

Supplementary Table 8

Original Data

## Data Availability

The bulk RNA-seq and scRNA-seq datasets, along with the corresponding analysis code, have been deposited in Synapse under accession numbers syn69911177, syn69911154, and syn74945338. All other data generated or analysed during this study are included in this published article and its supplementary information files.
