# Supplementary material for: Role of the ACTH-SF1/NR5A1-NRF2 axis in glucocorticoid-induced adrenocortical cell death through mitochondrial ROS accumulation
Source: Cell Death Dis. 2026 Oct 29;17(1):918. doi: 10.1038/s41419-026-09306-8 (PMC13720010; doi:10.1038/s41419-026-09306-8)
Supplement: Supplementary file 1 [file 41419_2026_9306_MOESM10_ESM.pdf]

Original Data

Full, uncropped Western blot images

Fig. 7F

KEAP1

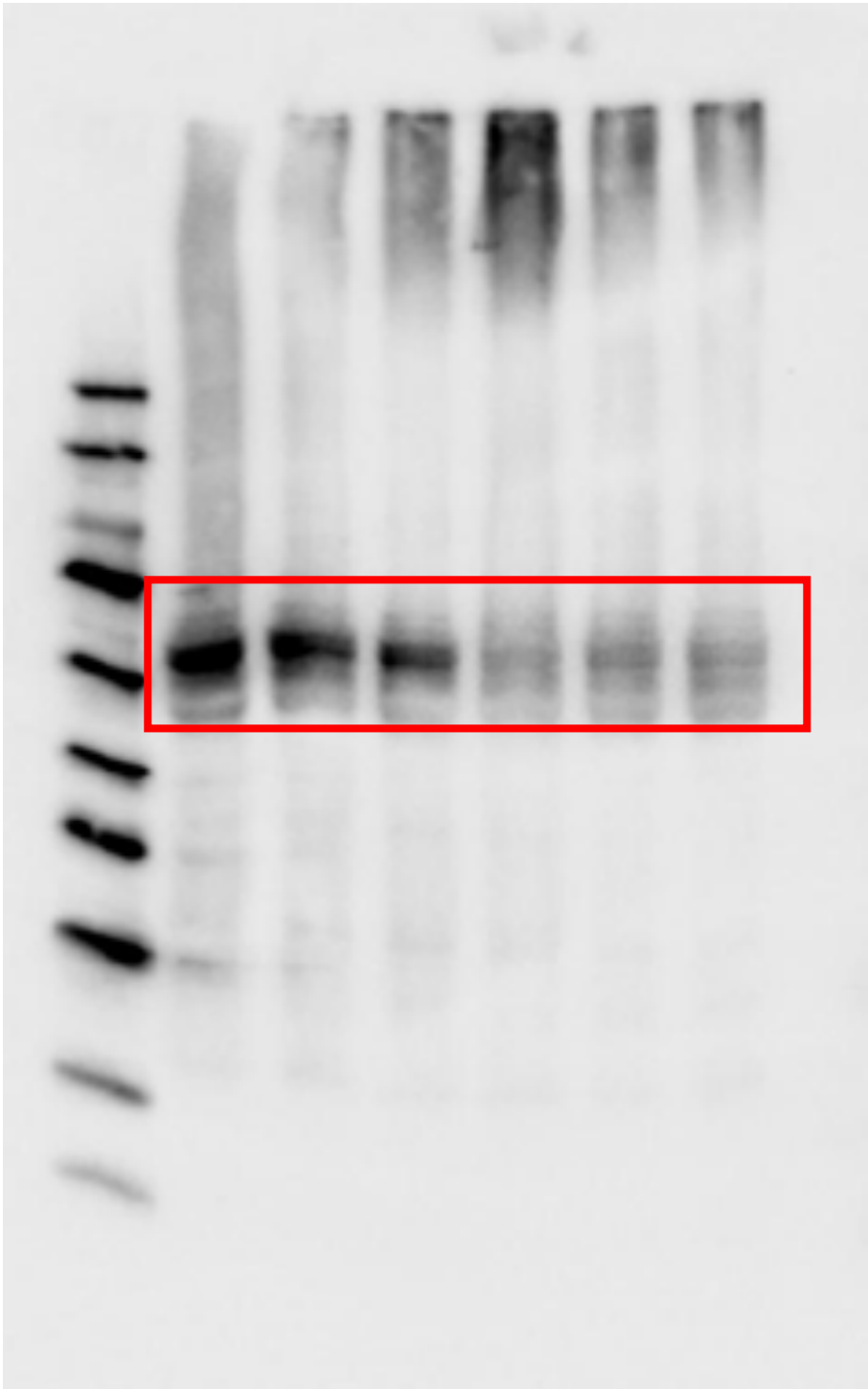

(#8047, Cell Signaling Technology)

GAPDH

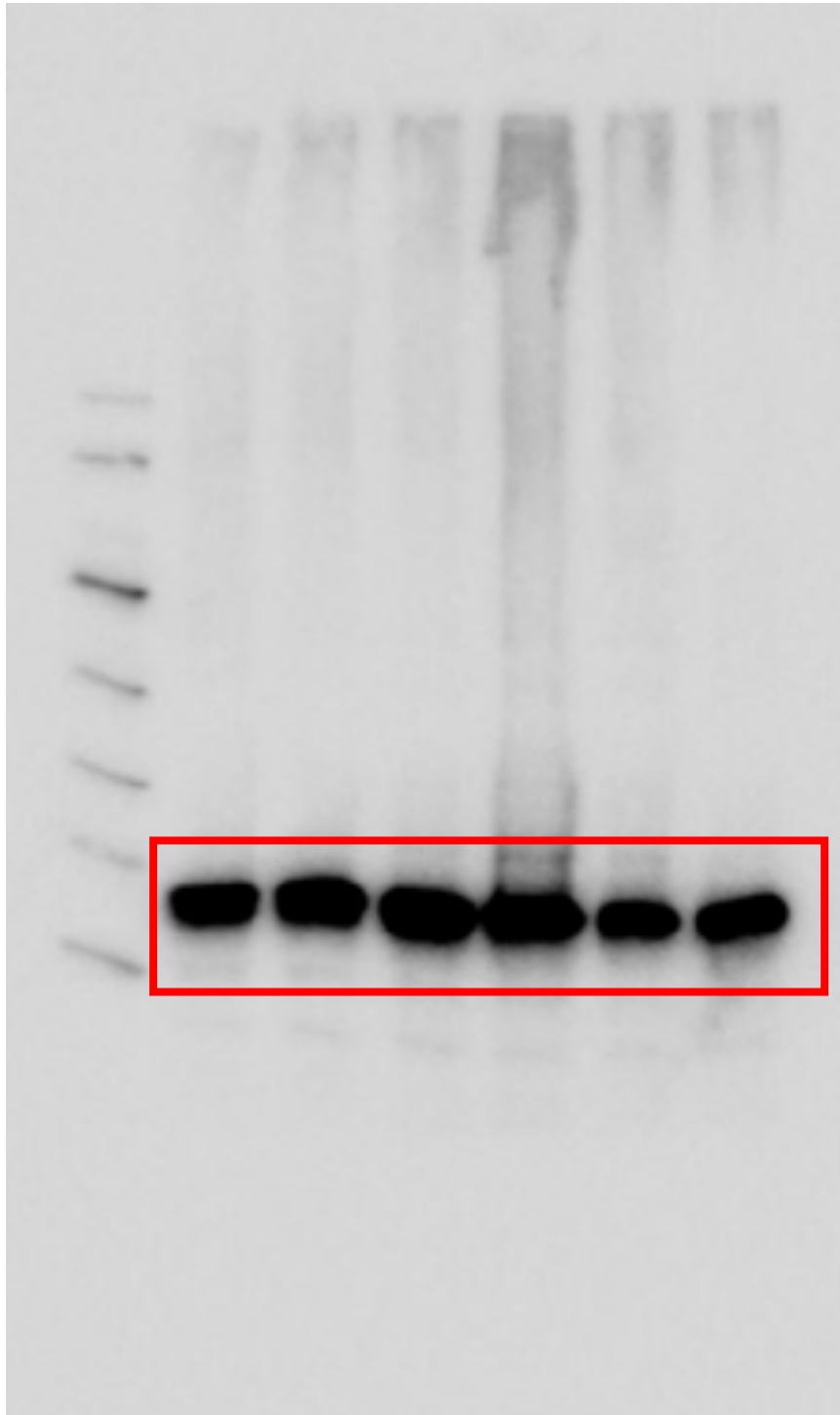

(#015-25473, FUJIFILM Wako Pure Chemical Corporation)

Fig. S3B

Phospho-eIF2 alpha

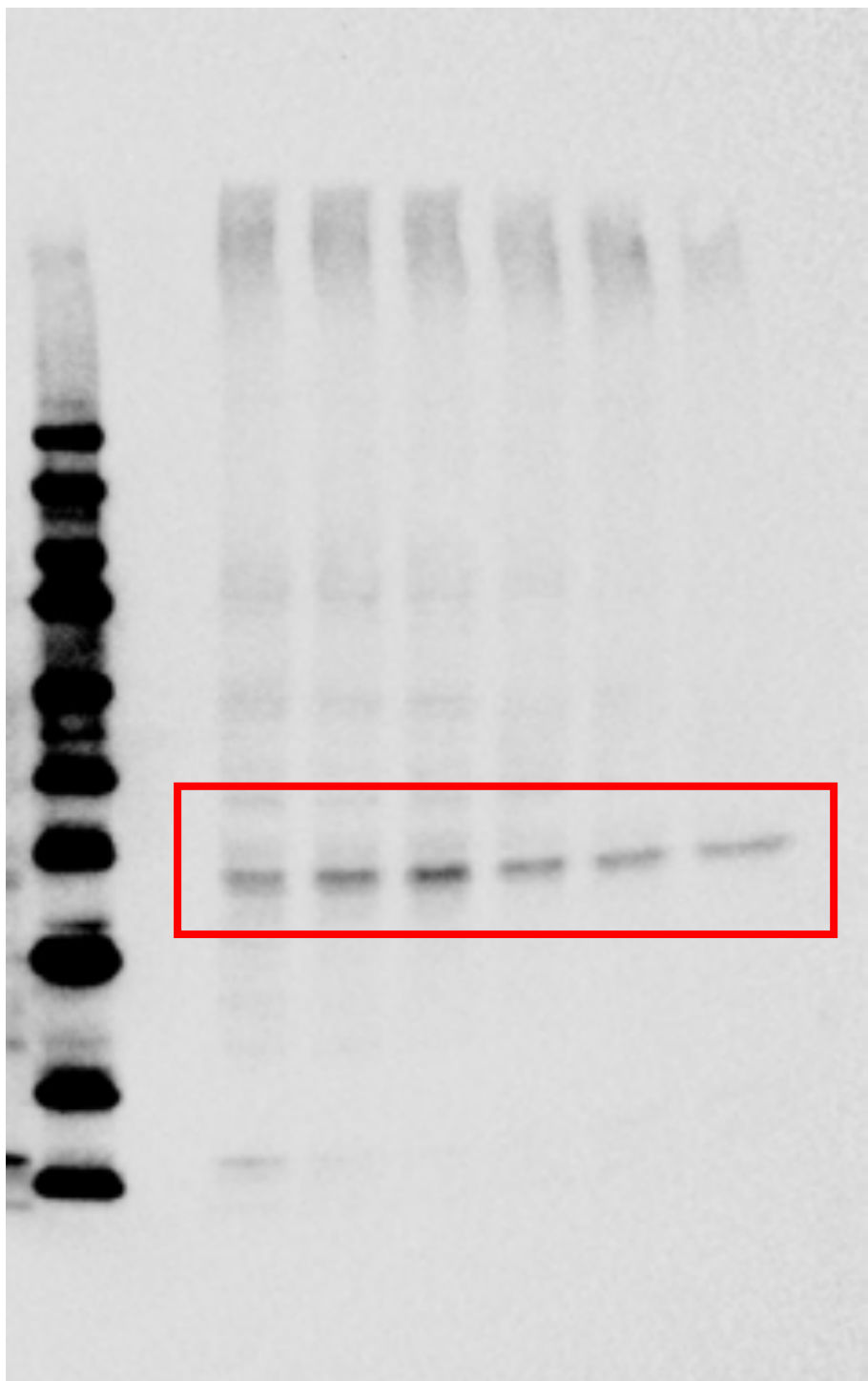

(#9721, Cell Signaling Technology)

eIF2 alpha

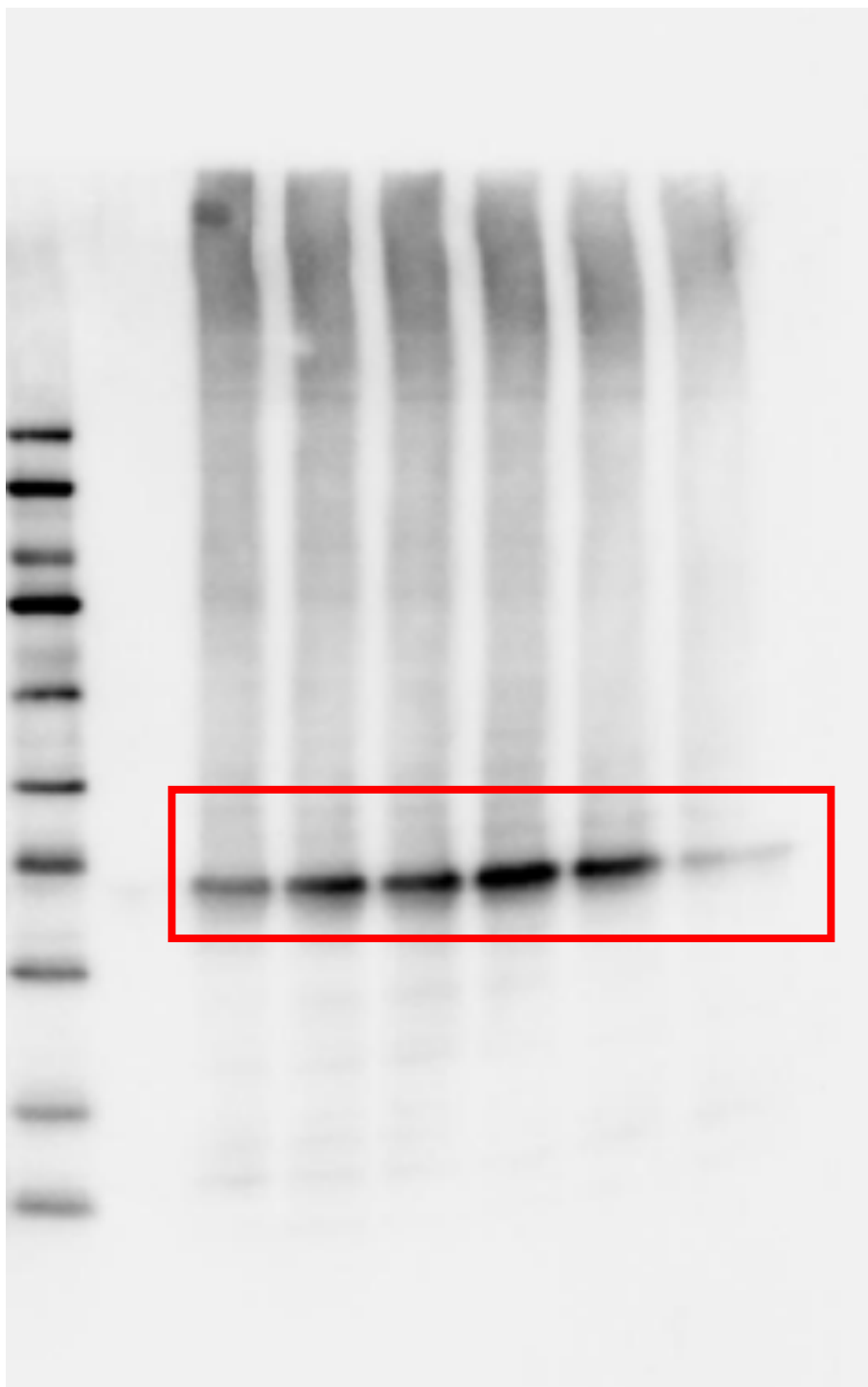

(#9722, Cell Signaling Technology)

RT-PCR data

Figure 3B

Cox1 mRNA expression, DEX treatment

Sample	Target	Ct
St-1	36b4	25.51679179
St-2	36b4	22.16457752
St-3	36b4	18.82851877
St-1	Cox1	20.74068448
St-2	Cox1	17.14710995
St-3	Cox1	13.93473798
Vehicle-1	36b4	18.60248717
Vehicle-2	36b4	19.26285988
Vehicle-3	36b4	19.29809571
Vehicle-4	36b4	18.91765853
Vehicle-5	36b4	18.87070543
Vehicle-6	36b4	18.67148003
DEX3-1	36b4	19.07931952
DEX3-2	36b4	19.08891786
DEX3-3	36b4	18.96943454
DEX3-4	36b4	18.95978676
DEX3-5	36b4	18.74784863
DEX3-6	36b4	18.85536719
DEX7-1	36b4	19.57931582
DEX7-2	36b4	20.07948016
DEX7-3	36b4	20.39132742
DEX7-4	36b4	18.88544991
DEX7-5	36b4	18.69771018
DEX7-6	36b4	18.93314056
DEX14-1	36b4	18.77176642
DEX14-2	36b4	18.78702566
DEX14-3	36b4	19.00264309
Vehicle-1	Cox1	13.88133973
Vehicle-2	Cox1	14.34260324
Vehicle-3	Cox1	14.4008527
Vehicle-4	Cox1	13.41534746
Vehicle-5	Cox1	13.88593129
Vehicle-6	Cox1	13.69472321
DEX3-1	Cox1	14.61835239
DEX3-2	Cox1	14.63816446
DEX3-3	Cox1	14.5683508
DEX3-4	Cox1	14.47942742
DEX3-5	Cox1	14.28637463
DEX3-6	Cox1	14.63147255
DEX7-1	Cox1	15.46574402
DEX7-2	Cox1	15.98407168
DEX7-3	Cox1	16.37973288
DEX7-4	Cox1	14.6356463
DEX7-5	Cox1	14.69395395
DEX7-6	Cox1	15.00366795
DEX14-1	Cox1	14.86629641
DEX14-2	Cox1	14.7779239
DEX14-3	Cox1	14.56985366

Cox2 mRNA expression, DEX treatment

Sample	Target	Ct
St-1	36b4	25.51679179
St-2	36b4	22.16457752
St-3	36b4	18.82851877
St-1	Cox2	34.06933797
St-2	Cox2	30.50860507
St-3	Cox2	27.3029715
Vehicle-1	36b4	18.60248717
Vehicle-2	36b4	19.26285988
Vehicle-3	36b4	19.29809571
Vehicle-4	36b4	18.91765853
Vehicle-5	36b4	18.87070543
Vehicle-6	36b4	18.67148003
DEX3-1	36b4	19.07931952
DEX3-2	36b4	19.08891786
DEX3-3	36b4	18.96943454
DEX3-4	36b4	18.95978676
DEX3-5	36b4	18.74784863
DEX3-6	36b4	18.85536719
DEX7-1	36b4	19.57931582
DEX7-2	36b4	20.07948016
DEX7-3	36b4	20.39132742
DEX7-4	36b4	18.88544991
DEX7-5	36b4	18.69771018
DEX7-6	36b4	18.93314056
DEX14-1	36b4	18.77176642
DEX14-2	36b4	18.78702566
DEX14-3	36b4	19.00264309
Vehicle-1	Cox2	24.39917912
Vehicle-2	Cox2	26.14437624
Vehicle-3	Cox2	27.12492395
Vehicle-4	Cox2	24.62416068
Vehicle-5	Cox2	25.00601154
Vehicle-6	Cox2	25.33375452
DEX3-1	Cox2	27.21814616
DEX3-2	Cox2	28.10702669
DEX3-3	Cox2	27.42076524
DEX3-4	Cox2	26.6182115
DEX3-5	Cox2	26.8916422
DEX3-6	Cox2	26.76996723
DEX7-1	Cox2	29.85130133
DEX7-2	Cox2	30.14005055
DEX7-3	Cox2	30.31770447
DEX7-4	Cox2	28.41774012
DEX7-5	Cox2	28.39384462
DEX7-6	Cox2	29.0110067
DEX14-1	Cox2	27.73987432
DEX14-2	Cox2	27.40423364
DEX14-3	Cox2	27.49537191

Figure 7A ①

Cyp11b1 mRNA expression, H-89 treatment

①

Sample	Target	Ct
St-1	36b4	24.20370481
St-2	36b4	20.39851116
St-3	36b4	17.20673185
St-1	Cyp11b1	22.01201433
St-2	Cyp11b1	18.24266397
St-3	Cyp11b1	14.89996993
Vehicle-1	36b4	16.98294036
Vehicle-2	36b4	17.08336424
Vehicle-3	36b4	16.78197899
H-89-1	36b4	16.33986187
H-89-2	36b4	16.32041773
H-89-3	36b4	16.37160303
Vehicle-1	Cyp11b1	26.84899248
Vehicle-2	Cyp11b1	26.68007836
Vehicle-3	Cyp11b1	26.27642841
H-89-1	Cyp11b1	27.92558239
H-89-2	Cyp11b1	27.86031872
H-89-3	Cyp11b1	27.06293199

②

Sample	Target	Ct
St-1	36b4	25.11688136
St-2	36b4	21.21940859
St-3	36b4	18.08025745
St-1	Cyp11b1	23.13270835
St-2	Cyp11b1	19.07101083
St-3	Cyp11b1	15.83816558
Vehicle-4	36b4	17.78194014
Vehicle-5	36b4	17.6137704
H-89-4	36b4	17.21661046
H-89-5	36b4	17.56909335
H-89-6	36b4	17.34390663
Vehicle-4	Cyp11b1	26.9870734
Vehicle-5	Cyp11b1	26.45066585
H-89-4	Cyp11b1	28.75977723
H-89-5	Cyp11b1	28.70939382
H-89-6	Cyp11b1	28.44681212

③

Sample	Target	Ct
St-1	36b4	26.34809851
St-2	36b4	21.73769167
St-3	36b4	18.42829296
St-1	Cyp11b1	24.86160278
St-2	Cyp11b1	19.60686663
St-3	Cyp11b1	16.08921492
Vehicle-6	36b4	17.88750675
Vehicle-7	36b4	18.03416233
Vehicle-8	36b4	17.87801929
H-89-7	36b4	18.00351981
H-89-8	36b4	18.01677749
Vehicle-6	Cyp11b1	28.26151775
Vehicle-7	Cyp11b1	28.12058409
Vehicle-8	Cyp11b1	27.75968865
H-89-7	Cyp11b1	28.19326064
H-89-8	Cyp11b1	28.0151508

④

Sample	Target	Ct
St-1	36b4	25.11688136
St-2	36b4	21.21940859
St-3	36b4	18.08025745
St-1	Cyp11b1	23.13270835
St-2	Cyp11b1	19.07101083
St-3	Cyp11b1	15.83816558
Vehicle-9	36b4	17.41442458
Vehicle-10	36b4	17.39450621
Vehicle-11	36b4	17.47593855
H-89-9	36b4	17.56718192
H-89-10	36b4	17.53709802
H-89-11	36b4	17.4837574
Vehicle-9	Cyp11b1	28.54241599
Vehicle-10	Cyp11b1	28.24803518
Vehicle-11	Cyp11b1	28.29935511
H-89-9	Cyp11b1	28.91072403
H-89-10	Cyp11b1	29.30862587
H-89-11	Cyp11b1	28.31279045

Figure 7A ②

Cyp11b1 mRNA expression, FSK treatment

①

Sample	Target	Ct
St-1	36b4	24.20370481
St-2	36b4	20.39851116
St-3	36b4	17.20673185
St-1	Cyp11b1	22.01201433
St-2	Cyp11b1	18.24266397
St-3	Cyp11b1	14.89996993
Vehicle-1	36b4	16.98294036
Vehicle-2	36b4	17.08336424
Vehicle-3	36b4	16.78197899
FSK-1	36b4	17.14175854
FSK-2	36b4	16.80643731
FSK-3	36b4	17.17484163
Vehicle-1	Cyp11b1	26.84899248
Vehicle-2	Cyp11b1	26.68007836
Vehicle-3	Cyp11b1	26.27642841
FSK-1	Cyp11b1	21.2889986
FSK-2	Cyp11b1	21.16392186
FSK-3	Cyp11b1	21.18556429

②

Sample	Target	Ct
St-1	36b4	25.11688136
St-2	36b4	21.21940859
St-3	36b4	18.08025745
St-1	Cyp11b1	23.13270835
St-2	Cyp11b1	19.07101083
St-3	Cyp11b1	15.83816558
Vehicle-4	36b4	17.78194014
Vehicle-5	36b4	17.6137704
FSK-4	36b4	17.79811359
FSK-5	36b4	17.81175073
FSK-6	36b4	17.89223449
Vehicle-4	Cyp11b1	26.9870734
Vehicle-5	Cyp11b1	26.45066585
FSK-4	Cyp11b1	20.76031277
FSK-5	Cyp11b1	20.801579
FSK-6	Cyp11b1	20.88528722

③

Sample	Target	Ct
St-1	36b4	26.34809851
St-2	36b4	21.73769167
St-3	36b4	18.42829296
St-1	Cyp11b1	24.86160278
St-2	Cyp11b1	19.60686663
St-3	Cyp11b1	16.08921492
Vehicle-6	36b4	17.88750675
Vehicle-7	36b4	18.03416233
Vehicle-8	36b4	17.87801929
FSK-7	36b4	18.00351981
FSK-8	36b4	18.01677749
FSK-9	36b4	18.12367773
Vehicle-6	Cyp11b1	28.26151775
Vehicle-7	Cyp11b1	28.12058409
Vehicle-8	Cyp11b1	27.75968865
FSK-7	Cyp11b1	22.62785547
FSK-8	Cyp11b1	22.37523796
FSK-9	Cyp11b1	22.48097829

Figure 7B

Nrf2 mRNA expression, H-89 treatment

①			②		
Sample	Target	Ct	Sample	Target	Ct
St-1	36b4	24.20370481	St-1	36b4	25.11688136
St-2	36b4	20.39851116	St-2	36b4	21.21940859
St-3	36b4	17.20673185	St-3	36b4	18.08025745
St-1	Nrf2	28.1801092	St-1	Nrf2	29.20606721
St-2	Nrf2	24.68405032	St-2	Nrf2	25.20880092
St-3	Nrf2	21.10848212	St-3	Nrf2	21.9404022
Vehicle-1	36b4	16.98294036	Vehicle-4	36b4	18.37465549
Vehicle-2	36b4	17.08336424	Vehicle-5	36b4	17.78194014
Vehicle-3	36b4	16.78197899	Vehicle-6	36b4	17.6137704
H-89-1	36b4	16.33986187	H-89-4	36b4	17.21661046
H-89-2	36b4	16.32041773	H-89-5	36b4	17.56909335
H-89-3	36b4	16.37160303	H-89-6	36b4	17.34390663
Vehicle-1	Nrf2	22.3306527	Vehicle-4	Nrf2	24.31438043
Vehicle-2	Nrf2	22.40628159	Vehicle-5	Nrf2	23.41385784
Vehicle-3	Nrf2	22.24149938	Vehicle-6	Nrf2	22.98157487
H-89-1	Nrf2	22.72971231	H-89-4	Nrf2	23.29955529
H-89-2	Nrf2	22.44159532	H-89-5	Nrf2	23.40290467
H-89-3	Nrf2	22.54727944	H-89-6	Nrf2	23.16750723

Gpx4 mRNA expression, H-89 treatment

①			②		
Sample	Target	Ct	Sample	Target	Ct
St-1	36b4	24.20370481	St-1	36b4	25.11688136
St-2	36b4	20.39851116	St-2	36b4	21.21940859
St-3	36b4	17.20673185	St-3	36b4	18.08025745
St-1	Gpx4	28.71182816	St-1	Gpx4	28.71182816
St-2	Gpx4	24.6795281	St-2	Gpx4	24.6795281
St-3	Gpx4	21.43249286	St-3	Gpx4	21.43249286
Vehicle-1	36b4	16.98294036	Vehicle-4	36b4	18.37465549
Vehicle-2	36b4	17.08336424	Vehicle-5	36b4	17.78194014
Vehicle-3	36b4	16.78197899	Vehicle-6	36b4	17.6137704
H-89-1	36b4	16.33986187	H-89-4	36b4	17.21661046
H-89-2	36b4	16.32041773	H-89-5	36b4	17.56909335
H-89-3	36b4	16.37160303	H-89-6	36b4	17.34390663
Vehicle-1	Gpx4	21.86239852	Vehicle-4	Gpx4	22.59438427
Vehicle-2	Gpx4	21.923899	Vehicle-5	Gpx4	22.07119101
Vehicle-3	Gpx4	21.63990702	Vehicle-6	Gpx4	21.58883387
H-89-1	Gpx4	21.85485838	H-89-4	Gpx4	21.40123748
H-89-2	Gpx4	21.89613169	H-89-5	Gpx4	21.64852534
H-89-3	Gpx4	21.91284891	H-89-6	Gpx4	21.75329297

Figure 7E

Nrf2 mRNA expression, FSK treatment

① Sample	Target	Ct	② Sample	Target	Ct
St-1	36b4	24.20370481	St-1	36b4	25.11688136
St-2	36b4	20.39851116	St-2	36b4	21.21940859
St-3	36b4	17.20673185	St-3	36b4	18.08025745
St-1	Nrf2	28.1801092	St-1	Nrf2	29.20606721
St-2	Nrf2	24.68405032	St-2	Nrf2	25.20880092
St-3	Nrf2	21.10848212	St-3	Nrf2	21.9404022
Vehicle-1	36b4	16.98294036	Vehicle-4	36b4	18.37465549
Vehicle-2	36b4	17.08336424	Vehicle-5	36b4	17.78194014
Vehicle-3	36b4	16.78197899	Vehicle-6	36b4	17.6137704
FSK-1	36b4	17.14175854	FSK-4	36b4	17.79811359
FSK-2	36b4	16.80643731	FSK-5	36b4	17.81175073
FSK-3	36b4	17.17484163	FSK-6	36b4	17.89223449
Vehicle-1	Nrf2	22.3306527	Vehicle-4	Nrf2	24.31438043
Vehicle-2	Nrf2	22.40628159	Vehicle-5	Nrf2	23.41385784
Vehicle-3	Nrf2	22.24149938	Vehicle-6	Nrf2	22.98157487
FSK-1	Nrf2	21.74558484	FSK-4	Nrf2	22.4822926
FSK-2	Nrf2	21.58937076	FSK-5	Nrf2	22.50124384
FSK-3	Nrf2	21.7550609	FSK-6	Nrf2	22.59418913

Gpx4 mRNA expression, FSK treatment

① Sample	Target	Ct	② Sample	Target	Ct
St-1	36b4	24.20370481	St-1	36b4	25.11688136
St-2	36b4	20.39851116	St-2	36b4	21.21940859
St-3	36b4	17.20673185	St-3	36b4	18.08025745
St-1	Gpx4	28.71182816	St-1	Gpx4	28.71182816
St-2	Gpx4	24.6795281	St-2	Gpx4	24.6795281
St-3	Gpx4	21.43249286	St-3	Gpx4	21.43249286
Vehicle-1	36b4	16.98294036	Vehicle-4	36b4	18.37465549
Vehicle-2	36b4	17.08336424	Vehicle-5	36b4	17.78194014
Vehicle-3	36b4	16.78197899	Vehicle-6	36b4	17.6137704
FSK-1	36b4	17.14175854	FSK-4	36b4	17.79811359
FSK-2	36b4	16.80643731	FSK-5	36b4	17.81175073
FSK-3	36b4	17.17484163	FSK-6	36b4	17.89223449
Vehicle-1	Gpx4	21.86239852	Vehicle-4	Gpx4	22.59438427
Vehicle-2	Gpx4	21.923899	Vehicle-5	Gpx4	22.07119101
Vehicle-3	Gpx4	21.63990702	Vehicle-6	Gpx4	21.58883387
FSK-1	Gpx4	21.61689042	FSK-4	Gpx4	21.30527332
FSK-2	Gpx4	21.32924375	FSK-5	Gpx4	21.31963808
FSK-3	Gpx4	21.57248978	FSK-6	Gpx4	21.39622728

Figure 8A ①

Sf1 mRNA expression, FSK treatment

①

Sample	Target	Ct
St-1	36b4	24.20370481
St-2	36b4	20.39851116
St-3	36b4	17.20673185
St-1	Sf-1	27.47186886
St-2	Sf-1	22.87792503
St-3	Sf-1	19.60022631
Vehicle-1	36b4	16.98294036
Vehicle-2	36b4	17.08336424
Vehicle-3	36b4	16.78197899
FSK-1	36b4	17.14175854
FSK-2	36b4	16.80643731
FSK-3	36b4	17.17484163
Vehicle-1	Sf-1	20.44634999
Vehicle-2	Sf-1	20.53241183
Vehicle-3	Sf-1	20.35745806
FSK-1	Sf-1	19.78190427
FSK-2	Sf-1	19.71322788
FSK-3	Sf-1	19.7826985

②

Sample	Target	Ct
St-1	36b4	25.11688136
St-2	36b4	21.21940859
St-3	36b4	18.08025745
St-1	Sf-1	27.34485857
St-2	Sf-1	23.66342954
St-3	Sf-1	20.47192436
Vehicle-4	36b4	17.78194014
Vehicle-5	36b4	17.6137704
FSK-4	36b4	17.79811359
FSK-5	36b4	17.81175073
FSK-6	36b4	17.89223449
Vehicle-4	Sf-1	21.5355096
Vehicle-5	Sf-1	21.22743663
FSK-4	Sf-1	20.21898195
FSK-5	Sf-1	20.36296615
FSK-6	Sf-1	20.38120216

③

Sample	Target	Ct
St-1	36b4	26.34809851
St-2	36b4	21.73769167
St-3	36b4	18.42829296
St-1	Sf-1	28.94571362
St-2	Sf-1	23.87765343
St-3	Sf-1	20.59753386
Vehicle-6	36b4	17.88750675
Vehicle-7	36b4	18.03416233
Vehicle-8	36b4	17.87801929
FSK-7	36b4	18.00351981
FSK-8	36b4	18.01677749
FSK-9	36b4	18.12367773
Vehicle-6	Sf-1	21.22808296
Vehicle-7	Sf-1	21.23141417
Vehicle-8	Sf-1	21.0075574
FSK-7	Sf-1	21.04911326
FSK-8	Sf-1	20.72964361
FSK-9	Sf-1	20.83324112

Figure 8A ②

Sf1 mRNA expression, H-89 treatment

①

Sample	Target	Ct
St-1	36b4	24.20370481
St-2	36b4	20.39851116
St-3	36b4	17.20673185
St-1	Sf-1	27.47186886
St-2	Sf-1	22.87792503
St-3	Sf-1	19.60022631
Vehicle-1	36b4	16.98294036
Vehicle-2	36b4	17.08336424
Vehicle-3	36b4	16.78197899
H-89-1	36b4	16.33986187
H-89-2	36b4	16.32041773
H-89-3	36b4	16.37160303
Vehicle-1	Sf-1	20.44634999
Vehicle-2	Sf-1	20.53241183
Vehicle-3	Sf-1	20.35745806
H-89-1	Sf-1	20.48840783
H-89-2	Sf-1	20.20773858
H-89-3	Sf-1	20.3758484

②

Sample	Target	Ct
St-1	36b4	25.11688136
St-2	36b4	21.21940859
St-3	36b4	18.08025745
St-1	Sf-1	27.34485857
St-2	Sf-1	23.66342954
St-3	Sf-1	20.47192436
Vehicle-4	36b4	17.78194014
Vehicle-5	36b4	17.6137704
H-89-4	36b4	17.21661046
H-89-5	36b4	17.56909335
H-89-6	36b4	17.34390663
Vehicle-4	Sf-1	21.5355096
Vehicle-5	Sf-1	21.22743663
H-89-4	Sf-1	21.02101175
H-89-5	Sf-1	21.09878344
H-89-6	Sf-1	21.01472349

③

Sample	Target	Ct
St-1	36b4	26.34809851
St-2	36b4	21.73769167
St-3	36b4	18.42829296
St-1	Sf-1	28.94571362
St-2	Sf-1	23.87765343
St-3	Sf-1	20.59753386
Vehicle-6	36b4	17.88750675
Vehicle-7	36b4	18.03416233
Vehicle-8	36b4	17.87801929
H-89-7	36b4	18.97294258
H-89-8	36b4	18.28227275
H-89-9	36b4	18.10686203
Vehicle-6	Sf-1	21.22808296
Vehicle-7	Sf-1	21.23141417
Vehicle-8	Sf-1	21.0075574
H-89-7	Sf-1	22.94988354
H-89-8	Sf-1	21.50635995
H-89-9	Sf-1	21.82745674

④

Sample	Target	Ct
St-1	36b4	25.11688136
St-2	36b4	21.21940859
St-3	36b4	18.08025745
St-1	Sf-1	30.10061462
St-2	Sf-1	23.65490661
St-3	Sf-1	20.48169632
Vehicle-9	36b4	17.41442458
Vehicle-10	36b4	17.39450621
Vehicle-11	36b4	17.47593855
H-89-10	36b4	17.56718192
H-89-11	36b4	17.53709802
H-89-12	36b4	17.4837574
Vehicle-9	Sf-1	21.16589474
Vehicle-10	Sf-1	21.18051173
Vehicle-11	Sf-1	21.12386538
H-89-10	Sf-1	21.1437254
H-89-11	Sf-1	21.32635115
H-89-12	Sf-1	21.21278332

Figure 8B ①

Sf1 mRNA expression, siSF1 transfection

①

Sample	Target	Ct
St-1	36b4	25.95956331
St-2	36b4	22.21323009
St-3	36b4	18.76158381
St-1	Sf1	27.73220494
St-2	Sf1	24.22225649
St-3	Sf1	20.97751018
Vehicle-1	36b4	18.37211388
Vehicle-2	36b4	19.16090243
Vehicle-3	36b4	18.33376734
siSF1-1	36b4	18.31053614
siSF1-2	36b4	18.14173578
siSF1-3	36b4	18.27152511
Vehicle-1	Sf1	21.7331448
Vehicle-2	Sf1	22.43121762
Vehicle-3	Sf1	21.60191622
siSF1-1	Sf1	22.51437661
siSF1-2	Sf1	22.59401441
siSF1-3	Sf1	22.66702057

②

Sample	Target	Ct
St-1	36b4	25.95956331
St-2	36b4	22.21323009
St-3	36b4	18.76158381
St-1	Sf1	27.73220494
St-2	Sf1	24.22225649
St-3	Sf1	20.97751018
Vehicle-4	36b4	18.45343849
Vehicle-5	36b4	18.3794484
Vehicle-6	36b4	18.42663332
Vehicle-7	36b4	18.41995727
siSF1-4	36b4	18.31896162
siSF1-5	36b4	18.5041327
siSF1-6	36b4	18.31843796
siSF1-7	36b4	18.36946142
siSF1-8	36b4	18.28053002
siSF1-9	36b4	18.40118955
Vehicle-4	Sf1	22.11255253
Vehicle-5	Sf1	22.12216455
Vehicle-6	Sf1	22.12024546
Vehicle-7	Sf1	22.04244074
siSF1-4	Sf1	22.78093112
siSF1-5	Sf1	23.15318875
siSF1-6	Sf1	22.503026
siSF1-7	Sf1	22.86147975
siSF1-8	Sf1	22.63706786
siSF1-9	Sf1	22.86356643

Figure 8B ②

Nrf2 mRNA expression, siSF1 transfection

①

Sample	Target	Ct
St-1	36b4	25.95956331
St-2	36b4	22.21323009
St-3	36b4	18.76158381
St-1	Nrf2	29.27839323
St-2	Nrf2	25.56559907
St-3	Nrf2	21.88887881
Vehicle-1	36b4	18.37211388
Vehicle-2	36b4	19.16090243
Vehicle-3	36b4	18.33376734
siSF1-1	36b4	18.31053614
siSF1-2	36b4	18.14173578
siSF1-3	36b4	18.27152511
Vehicle-1	Nrf2	22.83234451
Vehicle-2	Nrf2	23.38486294
Vehicle-3	Nrf2	22.61413834
siSF1-1	Nrf2	23.1283669
siSF1-2	Nrf2	22.75481134
siSF1-3	Nrf2	22.91169136

②

Sample	Target	Ct
St-1	36b4	25.95956331
St-2	36b4	22.21323009
St-3	36b4	18.76158381
St-1	Nrf2	29.27839323
St-2	Nrf2	25.56559907
St-3	Nrf2	21.88887881
Vehicle-4	36b4	18.45343849
Vehicle-5	36b4	18.3794484
Vehicle-6	36b4	18.42663332
Vehicle-7	36b4	18.41995727
siSF1-4	36b4	18.31896162
siSF1-5	36b4	18.5041327
siSF1-6	36b4	18.31843796
siSF1-7	36b4	18.36946142
siSF1-8	36b4	18.28053002
siSF1-9	36b4	18.40118955
Vehicle-4	Nrf2	23.31228786
Vehicle-5	Nrf2	23.40250043
Vehicle-6	Nrf2	23.23373559
Vehicle-7	Nrf2	23.30622916
siSF1-4	Nrf2	23.74847661
siSF1-5	Nrf2	24.08402994
siSF1-6	Nrf2	23.33096113
siSF1-7	Nrf2	23.7086428
siSF1-8	Nrf2	23.39447536
siSF1-9	Nrf2	23.6243142

Figure 8B ③

Gpx4 mRNA expression, siSF1 transfection

①

Sample	Target	Ct
St-1	36b4	25.95956331
St-2	36b4	22.21323009
St-3	36b4	18.76158381
St-1	Gpx4	29.12672346
St-2	Gpx4	25.31456275
St-3	Gpx4	21.76010785
Vehicle-1	36b4	18.37211388
Vehicle-2	36b4	19.16090243
Vehicle-3	36b4	18.33376734
siSF1-1	36b4	18.31053614
siSF1-2	36b4	18.14173578
siSF1-3	36b4	18.27152511
Vehicle-1	Gpx4	23.17426671
Vehicle-2	Gpx4	23.71292073
Vehicle-3	Gpx4	23.38198597
siSF1-1	Gpx4	23.62191376
siSF1-2	Gpx4	23.27657329
siSF1-3	Gpx4	23.39708324

②

Sample	Target	Ct
St-1	36b4	25.95956331
St-2	36b4	22.21323009
St-3	36b4	18.76158381
St-1	Gpx4	29.12672346
St-2	Gpx4	25.31456275
St-3	Gpx4	21.76010785
Vehicle-4	36b4	18.45343849
Vehicle-5	36b4	18.3794484
Vehicle-6	36b4	18.42663332
Vehicle-7	36b4	18.41995727
siSF1-4	36b4	18.31896162
siSF1-5	36b4	18.5041327
siSF1-6	36b4	18.31843796
siSF1-7	36b4	18.36946142
siSF1-8	36b4	18.28053002
siSF1-9	36b4	18.40118955
Vehicle-4	Gpx4	22.61241054
Vehicle-5	Gpx4	22.84846488
Vehicle-6	Gpx4	22.58750073
Vehicle-7	Gpx4	22.74705304
siSF1-4	Gpx4	22.65204539
siSF1-5	Gpx4	22.986734
siSF1-6	Gpx4	22.63790471
siSF1-7	Gpx4	22.89671096
siSF1-8	Gpx4	22.59364899
siSF1-9	Gpx4	22.75554784

Figure 8C ①

Sf1 mRNA expression, SF1 overexpression

Sample	Target	Ct
St-1	36b4	24.465
St-2	36b4	21.415
St-3	36b4	17.73
St-1	Sf1	27.035
St-2	Sf1	23.865
St-3	Sf1	20.295
Vehicle-1	36b4	19.065
Vehicle-2	36b4	18.285
Vehicle-3	36b4	18.495
Vehicle-4	36b4	18.73
Vehicle-5	36b4	18.14
Overexpression-1	36b4	17.605
Overexpression-2	36b4	18.41
Overexpression-3	36b4	17.435
Overexpression-4	36b4	18.235
Overexpression-5	36b4	18.04
Overexpression-6	36b4	18.275
Vehicle-1	Sf1	16.57
Vehicle-2	Sf1	16.135
Vehicle-3	Sf1	16
Vehicle-4	Sf1	16.11
Vehicle-5	Sf1	15.965
Overexpression-1	Sf1	13.395
Overexpression-2	Sf1	15.11
Overexpression-3	Sf1	13.83
Overexpression-4	Sf1	14.59
Overexpression-5	Sf1	14.595
Overexpression-6	Sf1	15.035

Nrf2 mRNA expression, SF1 overexpression

Sample	Target	Ct
St-1	36b4	24.465
St-2	36b4	21.415
St-3	36b4	17.73
St-1	Nrf2	27.975
St-2	Nrf2	24.74
St-3	Nrf2	21.145
Vehicle-1	36b4	19.065
Vehicle-2	36b4	18.285
Vehicle-3	36b4	18.495
Vehicle-4	36b4	18.73
Vehicle-5	36b4	18.14
Overexpression-1	36b4	17.605
Overexpression-2	36b4	18.41
Overexpression-3	36b4	17.435
Overexpression-4	36b4	18.235
Overexpression-5	36b4	18.04
Overexpression-6	36b4	18.275
Vehicle-1	Nrf2	25.15
Vehicle-2	Nrf2	23.68
Vehicle-3	Nrf2	24.115
Vehicle-4	Nrf2	24.36
Vehicle-5	Nrf2	23.25
Overexpression-1	Nrf2	23.315
Overexpression-2	Nrf2	23.51
Overexpression-3	Nrf2	22.535
Overexpression-4	Nrf2	23.165
Overexpression-5	Nrf2	23.275
Overexpression-6	Nrf2	23.22

Figure 8C ②

Gpx4 mRNA expression, SF1 overexpression

Sample	Target	Ct
St-1	36b4	25.98
St-2	36b4	22.05
St-3	36b4	17.97
St-1	Gpx4	29.22
St-2	Gpx4	25.01
St-3	Gpx4	21.21
Vehicle-1	36b4	23.27
Vehicle-2	36b4	22.77
Vehicle-3	36b4	24.38
Vehicle-4	36b4	24.37
Vehicle-5	36b4	23.92
Overexpression-1	36b4	23.65
Overexpression-2	36b4	23.78
Overexpression-3	36b4	23.41
Overexpression-4	36b4	24.09
Overexpression-5	36b4	23
Overexpression-6	36b4	24.33
Vehicle-1	Gpx4	28.42
Vehicle-2	Gpx4	27.79
Vehicle-3	Gpx4	28.9
Vehicle-4	Gpx4	29
Vehicle-5	Gpx4	28.13
Overexpression-1	Gpx4	27.84
Overexpression-2	Gpx4	27.8
Overexpression-3	Gpx4	27.41
Overexpression-4	Gpx4	28.12
Overexpression-5	Gpx4	26.83
Overexpression-6	Gpx4	28.06
