# Supplementary material for: Role of the ACTH-SF1/NR5A1-NRF2 axis in glucocorticoid-induced adrenocortical cell death through mitochondrial ROS accumulation
Source: Cell Death Dis. 2026 Oct 29;17(1):918. doi: 10.1038/s41419-026-09306-8 (PMC13720010; doi:10.1038/s41419-026-09306-8)
Supplement: Supplementary file 2 [file 41419_2026_9306_MOESM1_ESM.pdf]

Supplementary Figures

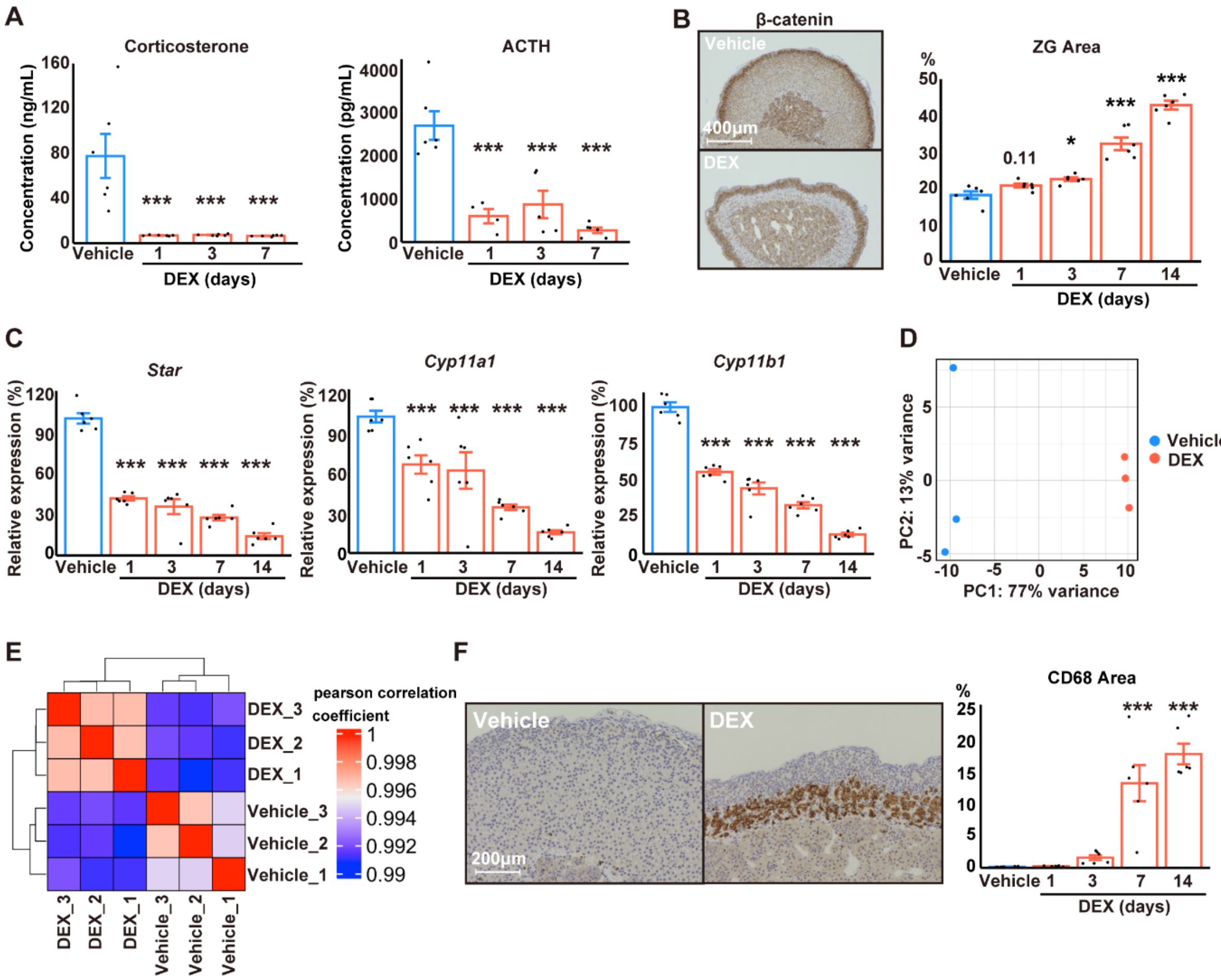

Figure S1. Dexamethasone (DEX) treatment suppresses steroidogenesis and promotes macrophage infiltration in the adrenal cortex.

(A) Plasma hormone concentrations measured by ELISA. Corticosterone levels are shown on the left, and adrenocorticotropic hormone (ACTH) levels on the right (n = 6 per group).

(B) Immunohistochemical (IHC) analysis for β-catenin to identify the zona glomerulosa (ZG). Representative images are shown on the left. The percentage of ZG area relative to the total cortical area is shown on the right (n = 6 per group).

(C) Gene expression of steroidogenic enzymes (*Star*, *Cyp11a1*, and *Cyp11b1*) in adrenal glands measured by reverse transcription quantitative polymerase chain reaction (RT-qPCR). Expression levels are shown relative to vehicle-treated mice.

(D) Principal component analysis (PCA) of transcriptomic profiles from DEX- and vehicle-treated mice based on RNA sequencing (RNA-seq) data.

(E) Pearson correlation coefficient matrix based on transcriptomic profiles of RNA-seq data from DEX- and vehicle-treated mice.

(F) IHC for CD68, a maker of macrophage, showing macrophage infiltration in the adrenal cortex. Representative images are shown on the left. The percentage of CD68-positive area relative to the cortical area is shown on the right (n = 6 per group).

All error bars represent SEM. *$P < 0.05$, ***$P < 0.001$ vs. vehicle-treated mice. Each data point represents an individual mouse.

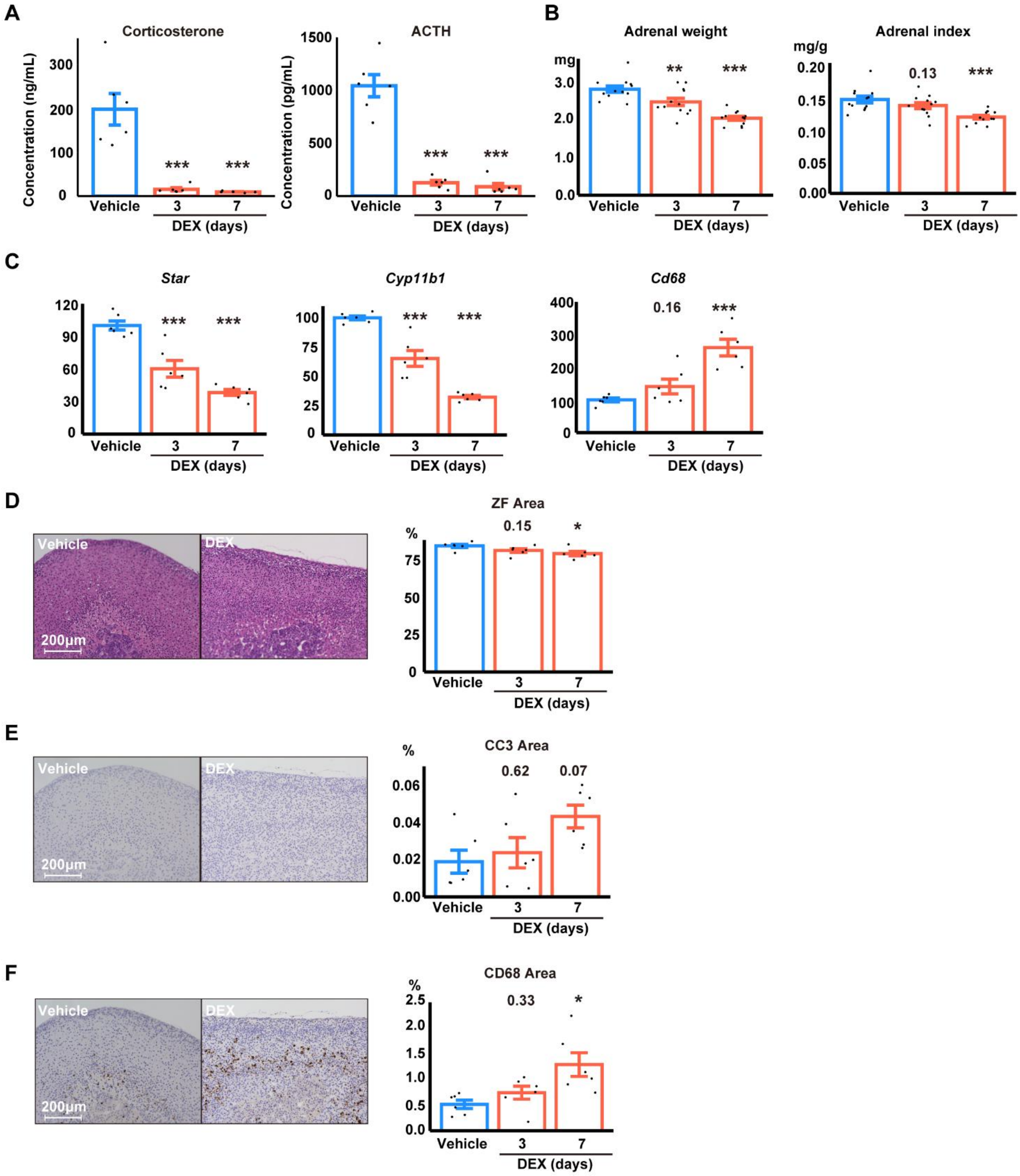

Figure S2. Effects of DEX treatment on adrenocortical alterations in female mice.

(A) Plasma hormone concentrations measured by ELISA. Corticosterone levels are shown on the left, and adrenocorticotropic hormone (ACTH) levels on the right (n = 6 per group). Each data point represents an individual mouse.

(B) Adrenal weight and adrenal index (adrenal weight/body weight) in DEX- and vehicle-treated mice (n = 6 per group; both adrenal glands per mouse were combined for analysis). Each data point represents an individual adrenal gland.

(C) Gene expression of *Star*, *Cyp11b1*, and *Cd68* in adrenal glands measured by RT-qPCR (n = 6 per group). Expression levels are shown relative to vehicle-treated mice. Each data point represents an individual mouse.

(D) HE staining of adrenal glands from DEX- and vehicle-treated mice. Representative images are shown on the left. Quantification of the ZF area (as a percentage of the total cortical area) is shown on the right (n = 6 per group). Each data point represents an individual mouse.

(E) IHC analysis for CC3 in the adrenal cortex from DEX- and vehicle-treated mice. Representative images are shown on the left. Quantification of CC3-positive area (as a percentage of the cortical area) is shown on the right (n = 6 per group). Each data point represents an individual mouse.

(F) IHC analysis for CD68 in the adrenal cortex from DEX- and vehicle-treated mice. Representative images are shown on the left. Quantification of CD68-positive area (as a percentage of cortical area) is shown on the right (n = 6 per group). Each data point represents an individual mouse.

All error bars represent SEM. *$P < 0.05$, **$P < 0.01$, ***$P < 0.001$ vs. vehicle-treated mice.

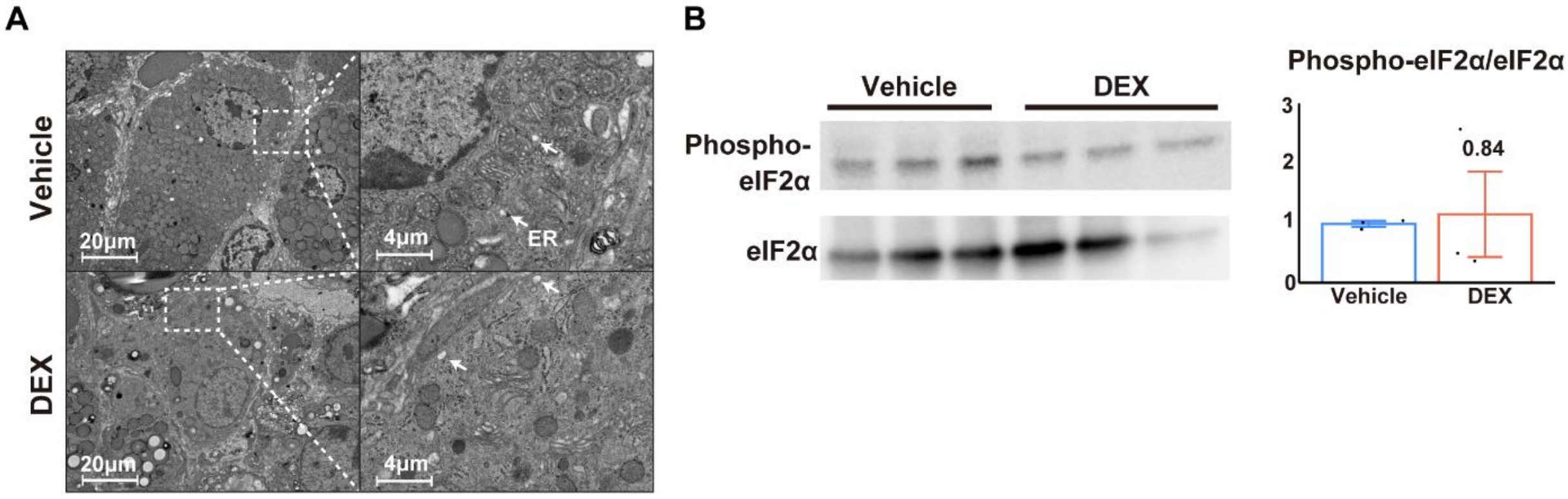

Figure S3. Effects of DEX treatment on endoplasmic reticulum (ER) stress in the mouse adrenal cortex.

(A) Representative TEM images of adrenal glands from 7 days DEX- and vehicle-treated mice. White arrows indicate ER. High resolution images are shown on the right.

(B) Western blot analysis of phosphorylated eIF2α and total eIF2α protein expression in adrenal glands from DEX- and vehicle-treated mice. Representative band images are shown on the left, and quantification of the phosphorylated eIF2α/total eIF2α ratio is shown on the right. All error bars represent SEM. Each data point represents an individual mouse.

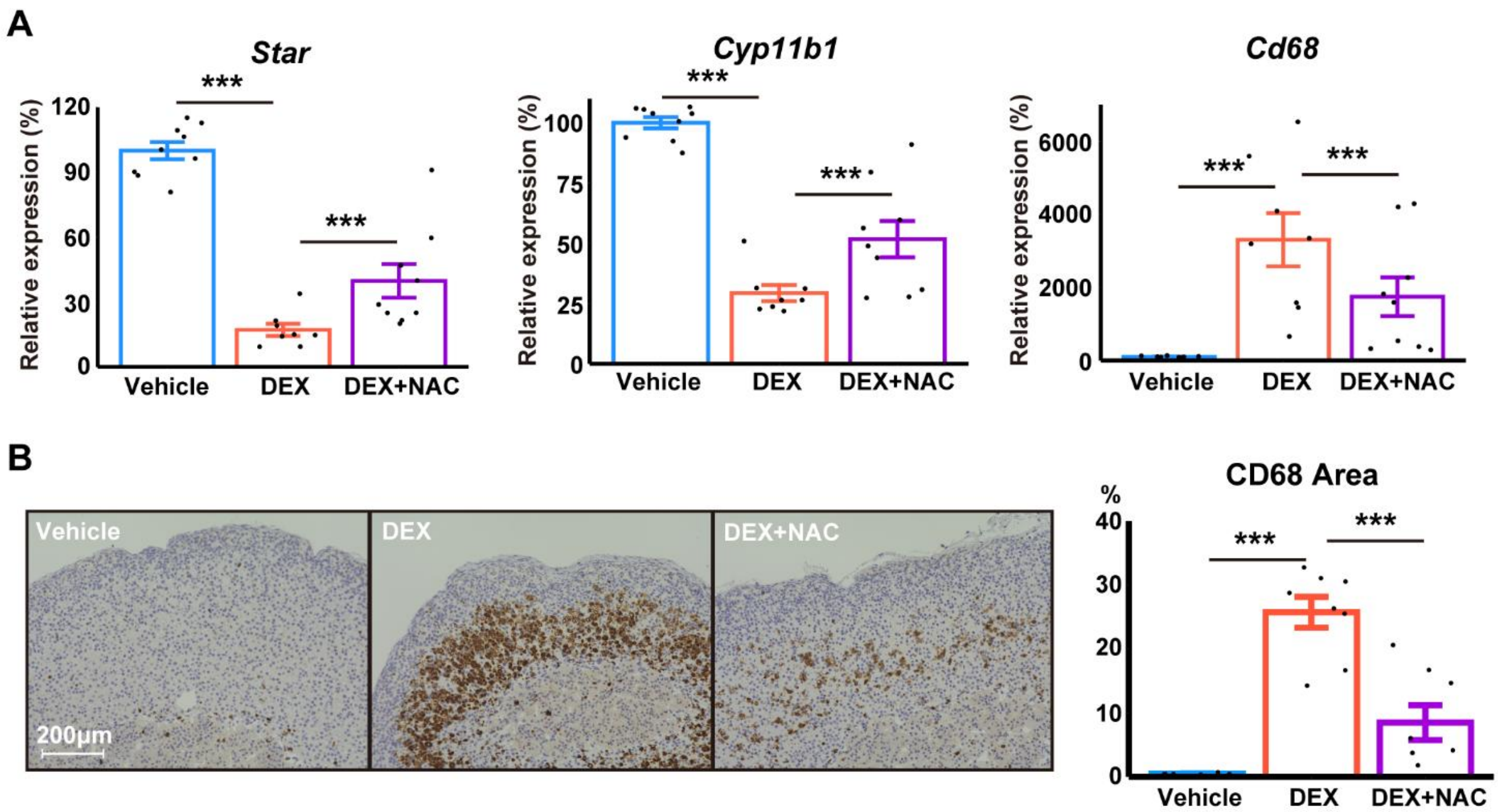

Figure S4. N-acetyl-L-cysteine (NAC) administration alleviates DEX-induced cell death and macrophage inflammation.

(A) Gene expression of *Star*, *Cyp11b1*, and *Cd68* in adrenal glands from DEX-, DEX+NAC-, and vehicle-treated mice, measured by RT-qPCR. Expression levels are shown relative to vehicle-treated mice (n = 8 per group).

(B) IHC for CD68 in the adrenal cortex. Representative images are shown on the left. Quantification of CD68-positive area (as a percentage of cortical area) is shown on the right (n = 8 per group).

All error bars represent SEM. ****P* < 0.001. Each data point represents an individual mouse.

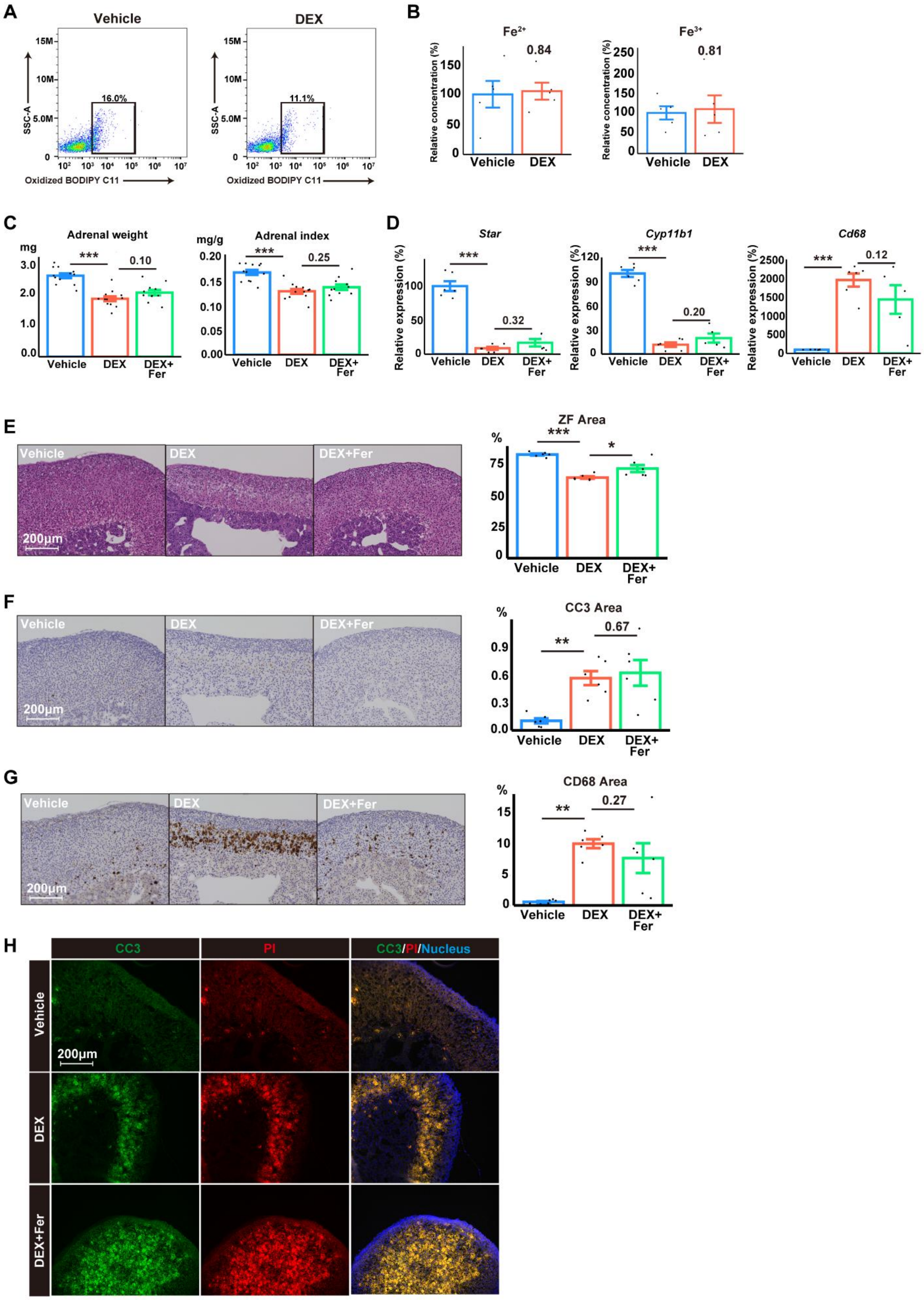
A
Vehicle
DEX
15M
10M
5.0M
0
SSC-A
16.0%
11.1%
Oxidized BODIPY C11
B
Fe2+
Fe3+
0.84
0.81
Relative concentration (%)
Vehicle
DEX
C
Adrenal weight
mg
***
0.10
Adrenal index
mg/g
***
0.25
Vehicle
DEX
DEX+
Fer
D
Star
Cyp11b1
Cd68
Relative expression (%)
***
0.32
***
0.20
***
0.12
E
ZF Area
%
***
*
200μm
Vehicle
DEX
DEX+Fer
F
CC3 Area
**
0.67
G
CD68 Area
**
0.27
H
CC3
PI
CC3/PI/Nucleus
Vehicle
DEX
DEX+Fer
200μm

Figure S5. Involvement of ferroptosis in DEX-induced adrenal dysfunction.

(A) Representative flow cytometry analysis of oxidized lipid peroxidation levels in isolated adrenocortical cells from DEX- and vehicle-treated mice. Cells were stained with BODIPY C11 and gated on singlets and live cells. Similar results were obtained in 3 independent experiments.

(B) Fe^{2+} and Fe^{3+} levels in the adrenal glands were measured using a colorimetric assay. Each value was normalized to the total protein content and is shown relative to the vehicle-treated group. Fe^{2+} levels are shown on the left, and Fe^{3+} levels are shown on the right. Each data point represents one pooled sample generated from 4 adrenal glands.

(C) Adrenal weight and adrenal index (adrenal weight/body weight) in DEX-, DEX+Ferrostatin-1 (Fer)-, and vehicle-treated mice (n = 6 per group; both adrenal glands per mouse were combined for analysis). Each data point represents an individual adrenal gland.

(D) Gene expression of *Star*, *Cyp11b1*, and *Cd68* in adrenal glands measured by reverse transcription quantitative polymerase chain reaction (RT-qPCR) (n = 6 per group). Expression levels are shown relative to vehicle-treated mice. Each data point represents an individual mouse.

(E) Hematoxylin and eosin (HE) staining of adrenal glands from DEX-, DEX+Fer-, and vehicle-treated mice. Representative images are shown on the left. Quantification of the ZF area (as a percentage of the total cortical area) is shown on the right. Each data point represents an individual mouse.

(F) IHC analysis for cleaved caspase-3 (CC3) in the adrenal glands from DEX-, DEX+Fer-, and vehicle-treated mice (n = 6 per group). Representative images are shown on the left. Quantification of CC3-positive area (as a percentage of the cortical area) is shown on the right (n = 6 per group). Each data point represents an individual mouse.

(G) IHC analysis for CD68 in the adrenal glands from DEX-, DEX+Fer-, and vehicle-treated mice (n = 6 per group). Representative images are shown on the left. Quantification of CD68-positive area (as a percentage of cortical area) is shown on the right (n = 6 per group).

(H) Representative images of immunofluorescence analysis of adrenal glands from DEX-, DEX+Fer-, and vehicle-treated mice. Green indicates CC3-positive cells, red indicates propidium iodide (PI)-positive cells, and blue indicates nucleus.

All error bars represent SEM. *$P < 0.05$, **$P < 0.01$, ***$P < 0.001$.

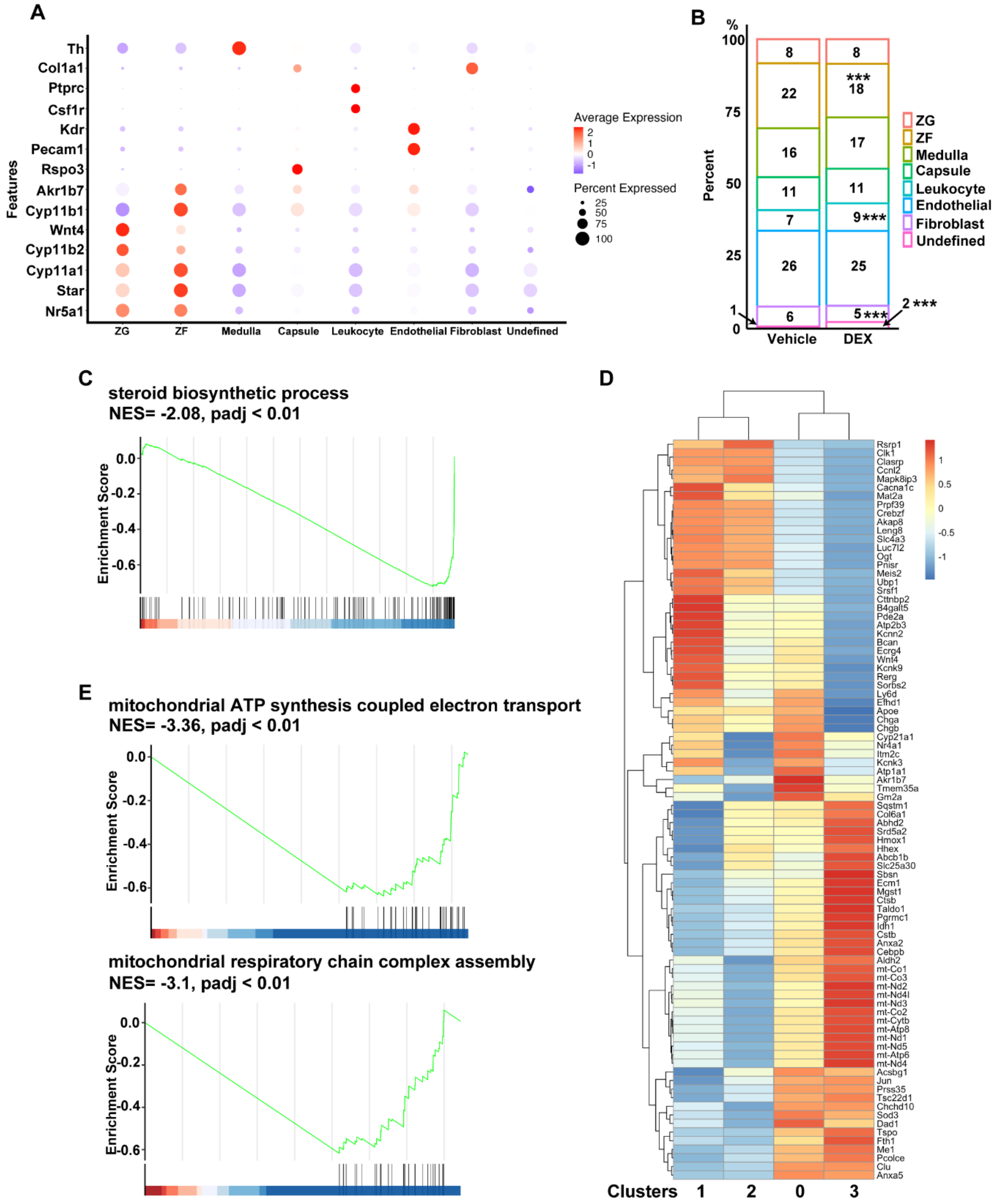

Figure S6. Single-cell transcriptomic profiling reveals downregulation of steroidogenic and mitochondrial pathways in zona fasciculata (ZF) cells following DEX treatment.

(A) Dot plot showing the expression of cell type–specific marker genes used for annotation of single-cell RNA seq clusters.

(B) Proportions of annotated adrenal cell types in DEX- and vehicle-treated mice. The proportions were analyzed using the chi-squared test, and statistical significance was determined based on adjusted *P* values. ***$P < 0.001$ vs. vehicle-treated mice.

(C) GSEA comparing ZF cells from DEX- and vehicle-treated mice. The positive NES indicates the enrichment of genes whose expression is upregulated in the DEX-treated adrenal glands in DEX-treated ZF cells. The steroid biosynthetic process is significantly downregulated in the DEX group.

(D) Heatmap displaying expression of differentially expressed genes (DEGs) across four re-clustered subpopulations of ZF cells.

(E) GSEA of transcriptomic data from cluster 2 in re-clustered ZF cells. The positive normalized enrichment score (NES) indicates the enrichment of genes whose expression is upregulated in cluster 2. Mitochondria-related pathways, including coupled electron transport and mitochondrial respiratory chain complex, are significantly downregulated.

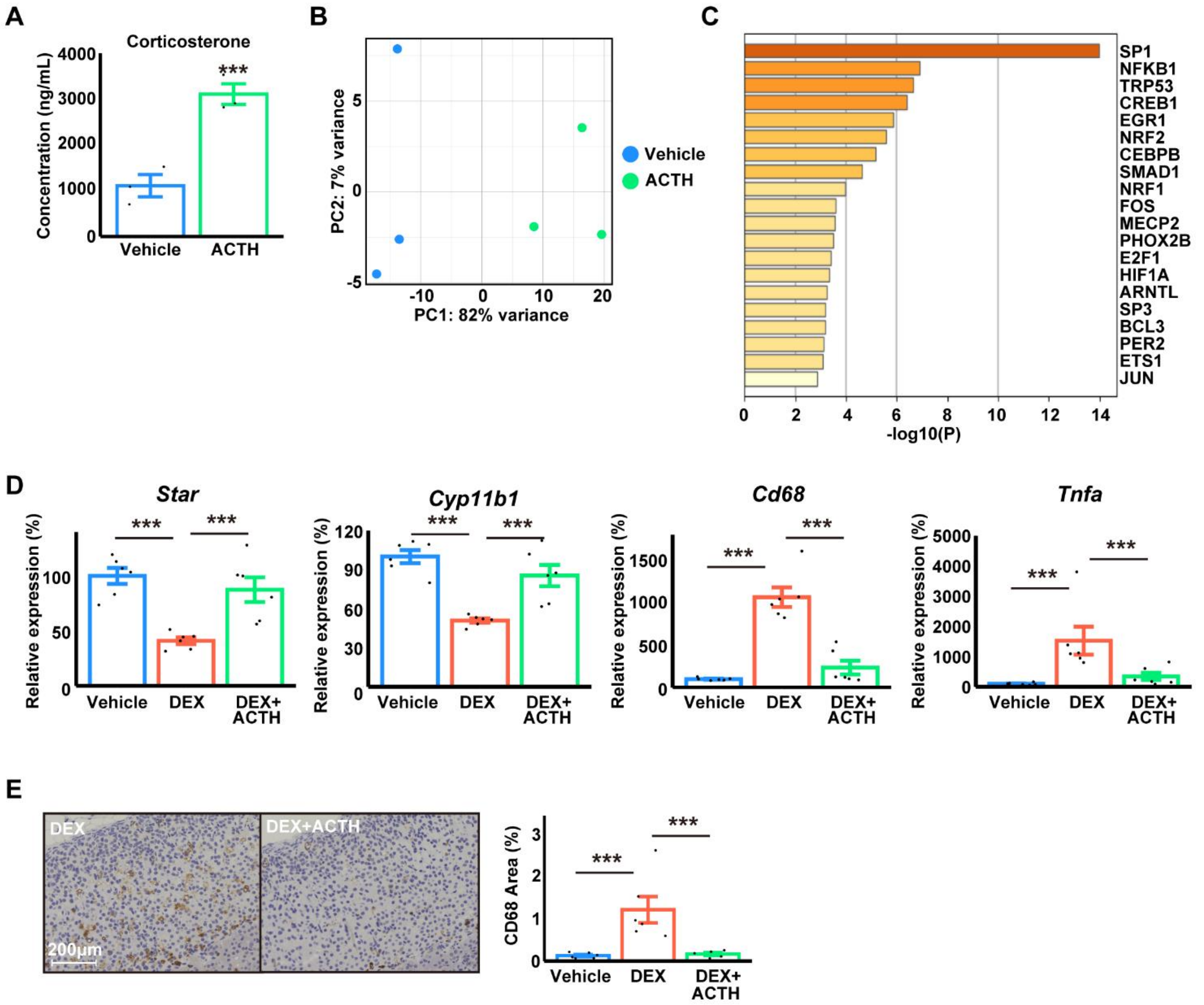

Figure S7. ACTH co-administration restores adrenocortical cell death and inflammation in the adrenal glands induced by DEX treatment.

(A) Plasma corticosterone concentrations measured by ELISA (n = 3 per group).

(B) Principal component analysis of transcriptomic profiles from adrenal glands of ACTH- and vehicle-treated mice based on RNA-seq data.

(C) Transcriptional Regulatory Relationships Unraveled by Sentence-based Text mining (TRRUST) analysis based on upregulated genes in ACTH-treated mice. Transcription factors predicted to be activated are ranked by statistical significance.

(D) Gene expression of *Star*, *Cyp11b1*, *Cd68*, and *Tnfa* in adrenal glands from DEX,

DEX+ACTH- and vehice-treated mice, measured by RT-qPCR. Expression levels are shown relative to vehicle-treated group (n = 6 per group).

(E) IHC for CD68 in the adrenal cortex. Representative images are shown on the left. Quantification of CD68-positive area (as a percentage of cortical area) is shown on the right (n = 6 per group).

All error bars represent SEM. ***$P < 0.001$. Each data point represents an individual mouse.
